# Retrotransposon-Induced Heterochromatin Spreading in the Mouse Revealed by Insertional Polymorphisms

**DOI:** 10.1371/journal.pgen.1002301

**Published:** 2011-09-29

**Authors:** Rita Rebollo, Mohammad M. Karimi, Misha Bilenky, Liane Gagnier, Katharine Miceli-Royer, Ying Zhang, Preeti Goyal, Thomas M. Keane, Steven Jones, Martin Hirst, Matthew C. Lorincz, Dixie L. Mager

**Affiliations:** 1 Terry Fox Laboratory, British Columbia Cancer Agency, Vancouver, Canada; 2 Department of Medical Genetics, University of British Columbia, Vancouver, Canada; 3 British Columbia Cancer Agency Genome Sciences Centre, Vancouver, Canada; 4 Wellcome Trust Sanger Institute, Hinxton, Cambridge, United Kingdom; Albert Einstein College of Medicine, United States of America

## Abstract

The “arms race” relationship between transposable elements (TEs) and their host has promoted a series of epigenetic silencing mechanisms directed against TEs. Retrotransposons, a class of TEs, are often located in repressed regions and are thought to induce heterochromatin formation and spreading. However, direct evidence for TE–induced local heterochromatin in mammals is surprisingly scarce. To examine this phenomenon, we chose two mouse embryonic stem (ES) cell lines that possess insertionally polymorphic retrotransposons (IAP, ETn/MusD, and LINE elements) at specific loci in one cell line but not the other. Employing ChIP-seq data for these cell lines, we show that IAP elements robustly induce H3K9me3 and H4K20me3 marks in flanking genomic DNA. In contrast, such heterochromatin is not induced by LINE copies and only by a minority of polymorphic ETn/MusD copies. DNA methylation is independent of the presence of IAP copies, since it is present in flanking regions of both full and empty sites. Finally, such spreading into genes appears to be rare, since the transcriptional start sites of very few genes are less than one Kb from an IAP. However, the *B3galtl* gene is subject to transcriptional silencing via IAP-induced heterochromatin. Hence, although rare, IAP-induced local heterochromatin spreading into nearby genes may influence expression and, in turn, host fitness.

## Author Summary

Transposable elements (TEs) are often thought to be harmful because of their potential to spread heterochromatin (repressive chromatin) into nearby sequences. However, there are few examples of spreading of heterochromatin caused by TEs, even though they are often found within repressive chromatin. We exploited natural variation in TE integrations to study heterochromatin induction. Specifically, we compared chromatin states of two mouse embryonic stem cell lines harboring polymorphic retrotransposons of three families, such that one line possesses a particular TE copy (full site) while the other does not (empty site). Nearly all IAP copies, a family of retroviral-like elements, are able to strongly induce repressive chromatin surrounding their insertion sites, with repressive histone modifications extending at least one kb from the IAP. This heterochromatin induction was not observed for the LINE family of non-viral retrotransposons and for only a minority of copies of the ETn/MusD retroviral-like family. We found only one gene that was partly silenced by IAP-induced chromatin. Therefore, while induction of repressive chromatin occurs after IAP insertion, measurable impacts on host gene expression are rare. Nonetheless, this phenomenon may play a role in rapid change in gene expression and therefore in host adaptive potential.

## Introduction

Transposable elements (TEs) are major constituents of eukaryotic genomes and are important catalysts of evolution [1], [2]. Indeed, TEs may cause negative, neutral or positive effects upon insertion, increase genomic instability by chromosomal rearrangements [3] and act as central collaborators in genome-wide regulatory network creation and renewal [4]. TEs are able to move throughout the genome either directly (DNA transposons) or by an RNA intermediate (Retrotransposons). Autonomous copies code for the necessary machinery for host invasion while non-autonomous copies will depend upon the former.

The well-known arms-race between TEs and the host genome [5] has resulted in several regulatory pathways, including a combination of various epigenetic mechanisms i.e. DNA methylation, small RNAs and histone post-translational modifications. In plants, invertebrate species and vertebrates, DNA methylation has been described as an important epigenetic silencing mechanism. In mouse, IAPs (Intracisternal A-type Particle elements), long terminal repeats (LTR) retrotransposons (also termed endogenous retroviruses (ERVs)), are highly DNA methylated and the disruption of enzymes responsible for such methylation (DNA methyltransferases Dnmt1 and Dnmt3L) causes global derepression of IAP copies [6], [7], albeit only in particular tissues. In *Arabidopsis thaliana,* TEs are strictly silenced by DNA methylation, which is often guided by small RNAs [8]. In *Drosophila melanogaster,* rasiRNAs and piRNAs (repeat-associated small interfering RNAs and piwi-interacting RNAs respectively) are responsible for the silencing of many TE copies [9], [10]. Small RNAs may trigger local heterochromatin [11] and histone post-translational modifications are also involved in TE silencing. Indeed, the repressive histone modifications H3K9me3 and H4K20me3 are associated with ERVs in mouse ES cells [12], [13]. Moreover, knock out of a histone methyltransferase (SETDB1) [14] or the protein that recruits it (KAP-1) [15] in mouse ES cells causes reduction of H3K9me3 at ERVs and induces high expression of several ERV classes as well as genes controlled by cryptic ERV promoters [16].

TEs are therefore often observed in regions associated with repressive histone marks and hence trapped into local heterochromatin. The analysis of euchromatin/heterochromatin boundaries has shown that an increase in TE density is co-localized with heterochromatin delimitation [17]. *D. melanogaster* mitotic chromosome analysis and the genome sequencing projects have also shown that TEs are abundant in heterochromatic regions [18], [19]. Such phenomena might be: 1. the consequence of insertional preferences of TEs into heterochromatin; 2. positive selection of TE maintenance into heterochromatin for genomic stability [20]; or 3. an induction of heterochromatin by TE sequences. The Sleeping beauty (SB) transposase has been shown to have an affinity for heterochromatin when transfected into mouse ES cells, however SB transposons do not seem to prefer heterochromatin over euchromatin [21]. In *Drosophila*, HeT-A, TAHRE and TART elements are found in telomeres but never in other heterochromatic regions, such as centromeres or interspersed heterochromatin, suggesting therefore an inclination for telomere specific sequences and not heterochromatin [22]. In general, no global heterochromatic insertional preference has been described for TEs with the exception of the yeast Ty5 retrotransposon that integrates into telomeres and the silent mating loci (the only two heterochromatic regions of the yeast genome) when the targeting domain of the Integrase is phosphorylated [23]. Some studies have found that only multiple tandem copies of a transgene (or even TEs) are able to induce heterochromatin but not a single copy [24]–[26]. Repetitive sequences are hypothesized and often claimed to induce heterochromatin and local region silencing [27], [28]. However, very few reports describe induction of heterochromatin at individual loci by a specific copy of a TE (see the sexual determination in melon by heterochromatin spreading from a DNA transposon for example [29]).

While genome-wide studies on histone post-translational modifications have revealed the repressive chromatin environment of several TE types in mouse ES cells [12], [16], no study has determined if TE insertion per se can induce the spreading of a repressive chromatin environment into flanking genomic regions. To determine if, indeed, TE families can induce local heterochromatin in a natural system, we surveyed two mouse strains where insertionally polymorphic TE copies have been documented [30], [31]. For the same genomic location, in the same cell type, we can distinguish a “full site” in one strain from an “empty site” in the other. Comparisons of the profiles of repressive epigenetic marks at both classes of sites allow us to determine the capacities of TEs to induce local heterochromatin. We report robust induction of H3K9me3-H4K20me3-chromatin spreading into nearby regions for the IAP family of LTR retrotransposons. Intriguingly, induction of such chromatin by other families of active LTR retrotransposons, including ETn/MusD (Early transposons), is much more variable. We found that transcription of one gene is impacted by the spread of IAP-induced heterochromatin in ES cells, but these effects on genes are likely rare, as such insertions are likely subject to strong negative selection.

## Results/Discussion

### Insertionally polymorphic TE families studied

We chose three different families of TEs for this study based on the data available on insertionally polymorphic copies, namely IAPs, ETn/MusD elements and LINEs (Long interspersed nuclear elements) [30], [31]. IAPs and ETn/MusDs are active families of mouse ERVs/LTR retrotransposons [30], together accounting for 10%–12% of spontaneous mutations in inbred mice [32]–[34]. ETn/MusD are a non-autonomous/autonomous pair of ERVs respectively [35], [36], where MusD appears to be more efficiently repressed by the host compared to their non-autonomous ETn cousins [37]. LINEs are non-LTR retrotransposons, abundant in the mouse genome with many active copies, although most are 5′ truncated due to their transposition mechanisms (dead-on-arrival copies) [38]. IAPs and ETn/MusD are highly associated with H4K20me3 and H3K9me3 in ES cells [12]–[14], [37] whereas LINEs are not associated with these marks [13]. No strong insertional biases have been described for these TE families apart from AT rich regions for LINEs [38], and analysis of the distribution of all three families, including common and polymorphic copies, reveals no obvious preference for heterochromatic regions or regions near genes (Figure S1).

### IAPs robustly induce heterochromatin

The use of our previous genome-wide analysis of H3K9me3 distribution in two ES cell lines (TT2 and J1) originating from different mouse backgrounds (C57Bl/6 x CBA F1 hybrid, 129S4/SvJae respectively) [16] allowed us to determine if TEs indeed induce local heterochromatin. Note that heterochromatin is herein defined according to the presence of the repressive histone post-translational modification H3K9me3 and H4K20me3. No significant differences have been observed in the overall load of TEs between B6 and 129 strains (our unpublished results). Two sets of copies were chosen: copies present in both ES cell lines (common copies) or copies present only in TT2 and absent in J1 cells (insertionally polymorphic copies of ETn/MusDs and IAPs). The inverse analysis, i.e. studying copies present in J1 and absent from TT2 was not performed since TT2 is a hybrid of CBA with B6 and our insertionally polymorphic data set does not include the CBA strain [30].

**Figure 1:**
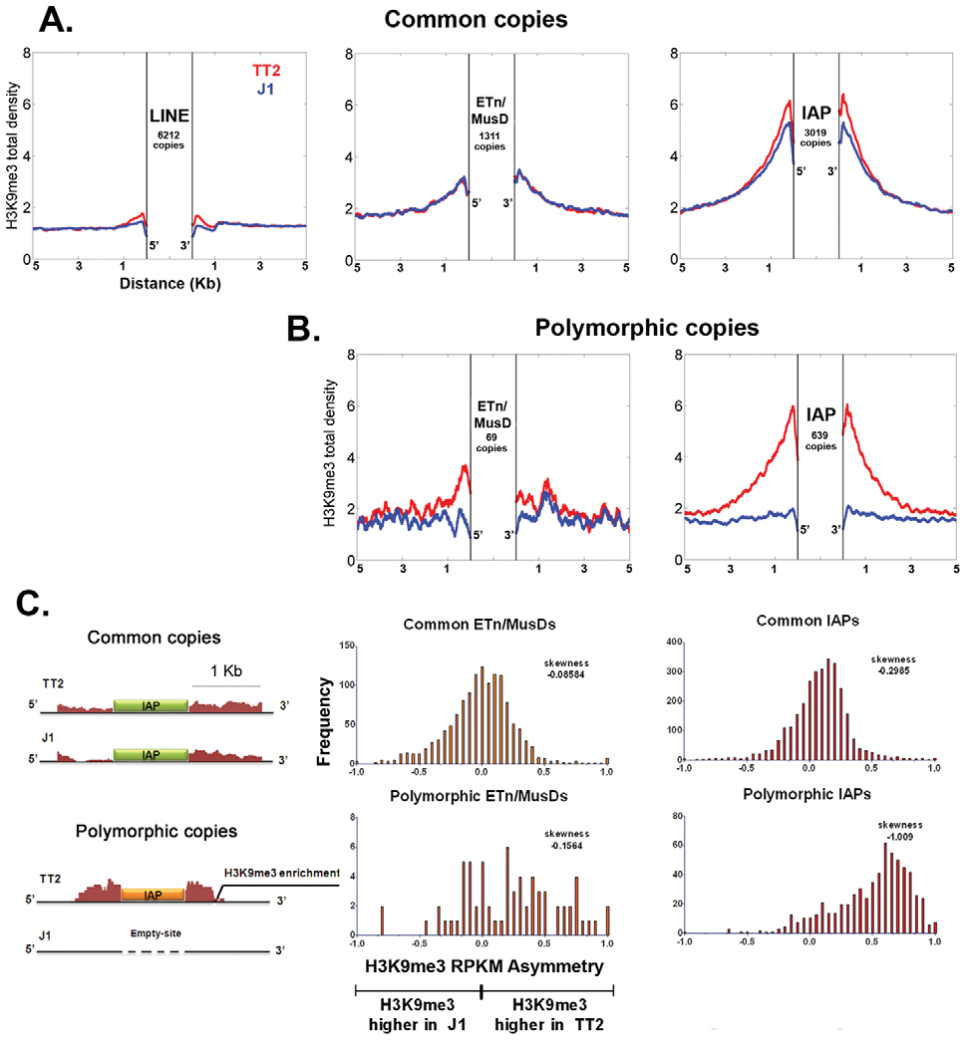
Detecting retrotransposon influence on H3K9me3 chromatin by comparison of common and insertionally polymorphic copies. A. H3K9me3 total density flanking common LINE (L1MdA), ETn/MusD and IAP copies in TT2 (red) and J1 (blue) cell lines. B. H3K9me3 total density flanking sites of insertionally polymorphic copies of ETn/MusDs and IAPs. Copies are present in TT2 (red) and absent in J1 (blue). C. RPKM asymmetry of H3K9me3 between TT2 and J1. A scheme shows an example of H3K9me3 RPKM comparison. Common copies (in green) and insertionally polymorphic copies (in orange) between both ES cell lines show different theoretical RPKM for H3K9me3 enrichment in the flanking regions (1 Kb – adapted from genome browser). Flanking regions harboring the same RPKM for both ES cell lines will have a RPKM asymmetry of 0 (illustrated with the common copy situation) while flanking regions having different RPKM will engender positive or negative RPKM asymmetries (illustrated with the polymorphic copy - present in TT2 and absent in J1). Note that no multi-mapping was allowed in our analysis creating a gap of H3K9me3 enrichment inside TE sequences themselves. Frequency is the number of copies having a given RPKM asymmetry. The skewness of each distribution is depicted.

Total average density of H3K9me3 was first calculated in regions flanking specific LINE, ETn/MusD and IAP copies present in both ES cell lines (common copies) (Figure 1A). It is important to note that the location of each copy was precisely known and the copies were present in the sequenced C57BL/6 reference genome [30], hence, all ChIP-seq reads matching the TE insertions themselves were excluded, allowing us to specifically examine the chromatin state of the flanking regions. Furthermore, since the ChIP-seq H3K9me3 data was generated using native-ChIP and the sequencing of DNA fragments were predominantly of mono-nucleosome size [16] (Figure S2), we could specifically observe the chromatin status of flanking regions with minimal background from H3K9me3 enriched TE copies. H3K9me3 is absent from the flanks of LINE elements (Figure 1A), consistent with previous analyses showing that these elements are themselves generally not marked by H3K9me3 [12], [13] (Figure S3A). However, ETn/MusDs and IAPs are associated with H3K9me3 enriched flanking regions in both ES cell lines, with the latter being particularly enriched (Figure 1A). Next we analyzed genomic loci harboring ERVs in TT2 cells but not in J1 cells (insertion site polymorphic copies) and observed that the average density of H3K9me3 is higher when an ETn/MusD or IAP is present, with IAPs again being most striking (Figure 1B). This analysis suggests that insertion of these ERVs causes deposition and spreading of this histone mark into flanking genomic DNA.

To determine if such tendency was a general phenomenon and not the result of only a few very enriched regions in TT2 cells (full site), we calculated the RPKM asymmetry (reads per kilobase per million mapped reads – see Materials and Methods) between flanking regions (1 Kb) of both strains, allowing us to distinguish regions differently enriched, i.e. regions where the RPKM asymmetries is near – 1 or +1 (see Figure 1C and Materials and Methods for data normalization and asymmetry calculation). Common copies of ETn/MusDs and IAPs show very similar marking of their flanking regions by H3K9me3 in both ES cell lines, as illustrated by a high frequency of copies near 0 RPKM asymmetry (Figure 1C and Figure S4). However, for polymorphic IAP copies, there is marked skewing towards high H3K9me3 in flanking regions of full sites (TT2) compared to empty sites (J1) (Figure 1C – skewness of -1.009). Hence, nearly all IAP copies induce local H3K9me3.

ETn/MusD elements show less pronounced skewing among polymorphic copies towards more H3K9me3 flanking full sites, but do show a different pattern when compared to common copies (Figure 1C). A minority of copies do indeed induce H3K9me3 deposition while the majority of the flanking regions do not seem to differ between full and empty sites. The limited number of polymorphic copies does not appear to be responsible for such a pattern since equivalently small sets of IAP polymorphic copies chosen randomly still show higher H3K9me3 in full sites (Figure S5). ETn/MusDs are highly expressed in ES cells (Figure S3A and S3B) and the copies identified by our study as being expressed (Figure S3 – note that uniquely aligned reads are biased towards old copies and therefore our analysis is an underrepresentation of expressed copies) are devoid of H3K9me3 and therefore do not promote spreading into the flanking regions. We have also observed ETn/MusD copies devoid of detectable expression and flanking H3K9me3 marking. We are unable to determine if individual IAP and ETn/MusD elements with equivalent levels of H3K9me3 are promoting different degrees of heterochromatin spreading into their flanking regions since the mappability of uniquely aligned reads is very low for single ERV copies (Figure S3C). Since the pattern of ETn/MusD H3K9me3 RPKM asymmetry is variable between copies it is possible that intrinsic characteristics of each copy may or may not trigger H3K9me3 deposition (see Figure S6 and Text S1).

### Other ERVs variably induce H3K9me3 chromatin

**Figure 2:**
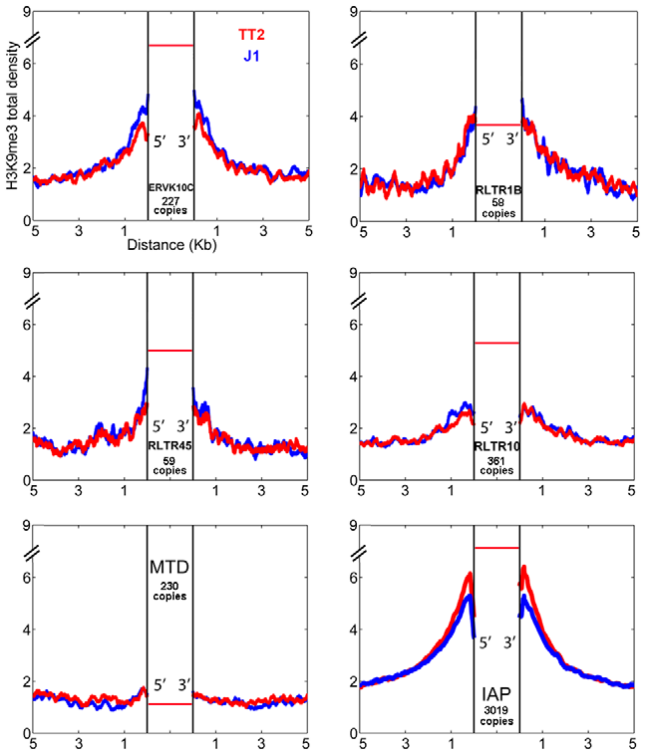
ERV influence on H3K9me3 chromatin. H3K9me3 total density flanking several ERV families in TT2 (red) and J1 (blue) cell lines. The total density of H3K9me3 was calculated on flanking regions (5′ and 3′) of ERVK10C, RLTR1B, RLTR45, RLTR10, MTD and IAPs (plot from Figure 1). All copies analyzed were full-length elements with two LTRs. For families other than IAP, copies are present in B6 (TT2, blue) but their presence in the CBA (TT2) and 129 strains (J1, red) is unknown. The red horizontal bar represents the H3K9me3 enrichment per copy (see also Figure S3A).

Since very different scenarios were observed for ETn/MusDs and IAPs, we asked if other ERVs could also induce H3K9me3 chromatin in their flanking regions. We analyzed the flanking regions of full-length elements of four ERV families known to be regulated by H3K9me3 and one family (MTD) lacking H3K9me3 [16]. Note that we do not have insertional polymorphic data for these families but the vast majority are expected to be present in both J1 and TT2 since there is little evidence for recent retrotranspositional activity as judged by ERV-induced germ line mutations [34]. Most ERVs marked and regulated by H3K9me3 [16] do spread this mark into flanking regions but again, to different degrees (Figure 2). ERVK10C and RLTR1B robustly induce H3K9me3 while RLTR45 and RLTR10 present a modest enrichment, and MTD elements, as expected, are not associated with H3K9me3 in the flanking regions. Apart from ETn/MusD, the ERV families studied show low levels of overall expression (Figure S3A). However, analysis at the level of individual copies reveals that the few copies that are expressed are devoid of H3K9me3 (Figure S3B). As the percentage of expressed ERVs within each of these ERV families (as well as LINE elements) is low in ES cells, no correlation can be drawn between their expression and the different degrees of H3K9me3 spreading into their flanking regions. The presence of high H3K9me3 enrichment within the ERV sequences themselves is observed for all ERVs with the exception of MTD (Figure S3A). However, due to the mappability limitation (Figure S3C), we are unable to determine if single ERV copies marked with H3K9me3 from different families equally promote spreading towards flanking regions. Therefore, while regulation by H3K9me3 seems correlated with deposition of this mark in the flanks, it does not correlate with the robustness or level of such deposition, which seems to be either ERV-specific and/or single copy specific. Out of all the TEs analyzed, IAPs induce the highest level of H3K9me3 enrichment in their nearby flanking sequences.

### Characterization of IAP-induced H3K9me3 chromatin

**Figure 3:**
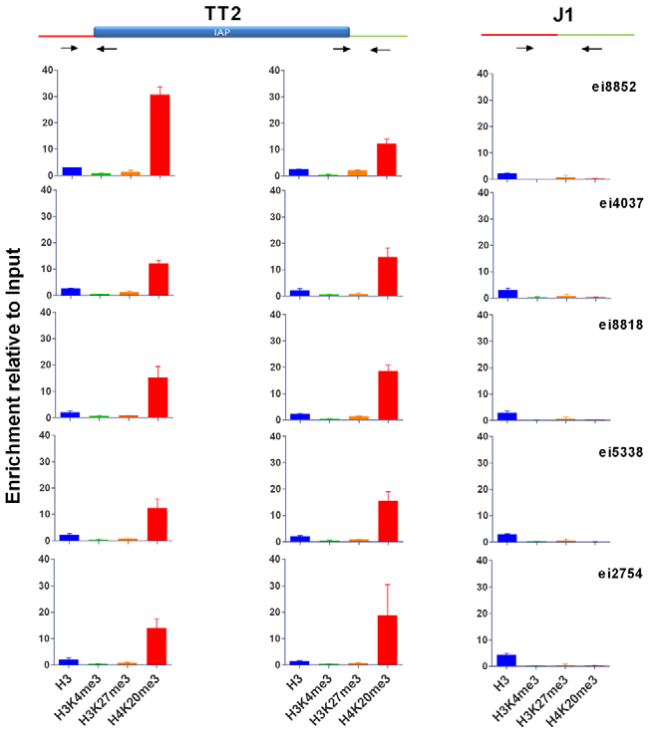
Characterization of IAP-induced chromatin by ChIP-qPCR. Chromatin immunoprecipitation followed by qPCR was done in TT2 (full sites) and J1 (empty sites). Enrichment for all antibodies was tested with positive and negative controls (Figure S10). Arrows represent the primers used for qPCR in both cell lines, where one primer is in the flanking region and the other in the IAP copy. For primers located further away from the IAP see Figure S9. Enrichment is shown as relative to the Input samples and the mean of the two biological replicates is depicted with the standard deviation.

To better characterize the chromatin induced by IAPs, we chose five copies in neighborhoods devoid of genes and if possible other repeats in order to observe an unbiased environment far from potential selective pressures (Figure S7). We used genomic PCR to confirm the presence of IAP copies in both alleles of the TT2 cells (C57BL/6 x CBA F1 hybrid) and their absence in J1 cells (129 origin) (Table 1). We also confirmed the presence of H3K9me3 in their flanking regions (Figure S8) and assayed for the presence of H3K4me3 (permissive modification), H3K27me3 (repressive, but observed in bivalent domains together with H3K4me3 in ES cells [39]) and H4K20me3 (repressive) (Figure 3 and Figure S9, see Figure S10 for ChIP controls). The only histone post-translational modification that spreads into genomic DNA flanking IAP copies together with H3K9me3 is H4K20me3. These post-translational modifications often target the same regions and were shown previously to be associated with IAPs and LTR retrotransposons in general [12]–[14]. All other marks analyzed are absent from both the empty and full sites. It is important to note that Histone 3 is equally present in both sites, eliminating the possibility that full sites have more nucleosomes and hence are more enriched in histone modifications than empty sites (Figure 3). Polycomb group proteins (PcG) mediate the methylation of Histone 3 at lysine 27 [40]. Knock out of both PcG complexes induces loss of DNA methylation and upregulation of IAP copies [41]. Nevertheless no specific association of H3K27me3 with IAP was observed in our study which may be a consequence of our choice of copies being far from genes. Alternatively, induction of IAP expression in PcG depleted cells may be the result of indirect effects.

**Table 1:** Confirmation of IAP presence.

				Presence of IAP by PCR			
IAP#	Chr	Start	End	129	J1	TT2	B6
1: 8852	14	72200881	72203815	−/−	−/−	+/+	+/+
2: 8818	14	71618515	71622436	−/−	−/−	+/+	+/+
3: 5338	5	41208811	41213676	−/−	−/−	+/+	+/+
4: 4037	15	33588765	33591413	−/−	−/−	+/+	+/+
5: 2754	2	11964849	11968035	−/−	−/−	+/+	+/+
1072970530(B3galtl)	5	150480464	…	+/+	+/+	−/−	−/−

The presence of an IAP copy for the five studied cases and the IAP near the *B3galtl* gene was confirmed by PCR in both ES cell lines (TT2 and J1) and also in the pure strains (129 and B6). Numbers in column one refer to IAP copy ID [30]. Chr: chromosome. +: presence; -: absence; +/+: homozygous. Coordinates refer to the sequenced C57BL/6 (B6) mouse genome (mm9).

**Figure 4:**
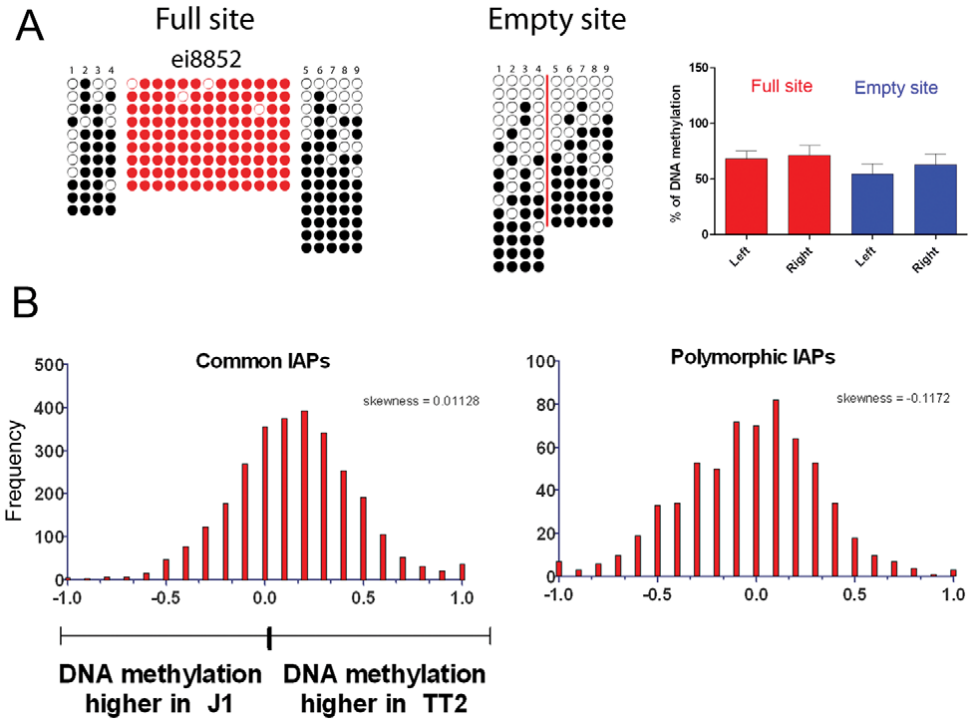
DNA methylation analysis of IAPs in both ES cell lines. A. Bisulfite conversion was done on DNA from both TT2 (full site) and J1 (empty site) cell lines and PCR followed by sequencing was done for flanking regions in J1, and LTR and flanking regions for TT2. Dark circles are methylated CpGs and empty circles are unmethylated CpGs. Red-colored circles are CpGs within the IAP LTRs and numbered CpGs between full and empty sites correspond to the same CpG site. A red line is depicted in the empty site illustrating the position of the IAP copy in the full site. Only one copy is depicted in this figure, with the remaining 4 copies shown in Figure S11. B. RPKM asymmetry of DNA methylation as measured by MeDIP on flanking regions between TT2 and J1. Common copies are present in both cell lines, polymorphic copies are only present in TT2. See Materials and Methods for asymmetry and skewness calculations.

As described above, silencing of TEs is often associated with DNA methylation. It is important to note that 70–80% of all CpGs in the mouse genome are methylated, half in repeats [42]. CpG Islands seem to be the primary exception, as they generally remain unmethylated [43], [44]. This fact increases the probability that TEs insert into DNA methylated regions. Bisulfite sequencing of the five flanking regions of both full sites and empty sites show methylation regardless of the presence of an IAP copy (Figure 4A and Figure S11). A significant increase in DNA methylation compared to the empty site was observed on only one side of two copies (Figure S11). To obtain a global view of DNA methylation status, we performed a genome wide DNA methylation analysis (MeDIP-seq) in TT2 and J1 cells. No significant difference was observed between full and empty sites, in agreement with our bisulfite data (Figure 4B).

The presence of IAP copies is therefore not necessary for DNA methylation of the flanking regions analyzed. Also, together with many other reports [6], [45], [46] we show that IAP copies are indeed methylated (Figure 4A and Figure S11 – red circles).

### Spreading of H3K9me3 associated with IAP insertion

Spreading of heterochromatin was first described in *Drosophila melanogaster* as a phenomenon called “position effect variegation” (PEV) where a transgene may be silenced if near heterochromatin (for a review see [47]). However, there are few documented examples of spreading of heterochromatin into flanking sequences and genes from TEs. In one case, two mouse B1 sequences were described as playing a crucial role in the establishment of a specific DNA methylation signal which appeared to be spreading towards flanking sequences but not reaching the nearby gene *Aprt* [48]. In mouse ES cells, 78% of sites comprising both H3K9me3 and H4K20me3 are near a satellite repeat or an IAP/ETn copy (maximum distance of 2Kb) [12]. In plants, S1 retrotransposons may lead to DNA methylation spreading into flanking sequences [49]. Finally, the most interesting and well-documented case of spreading of DNA methylation was also observed in plants, in which a DNA transposon is responsible for the spreading of DNA methylation into the *CmWIP1* promoter leading to sexual determination in the melon [29].

**Figure 5:**
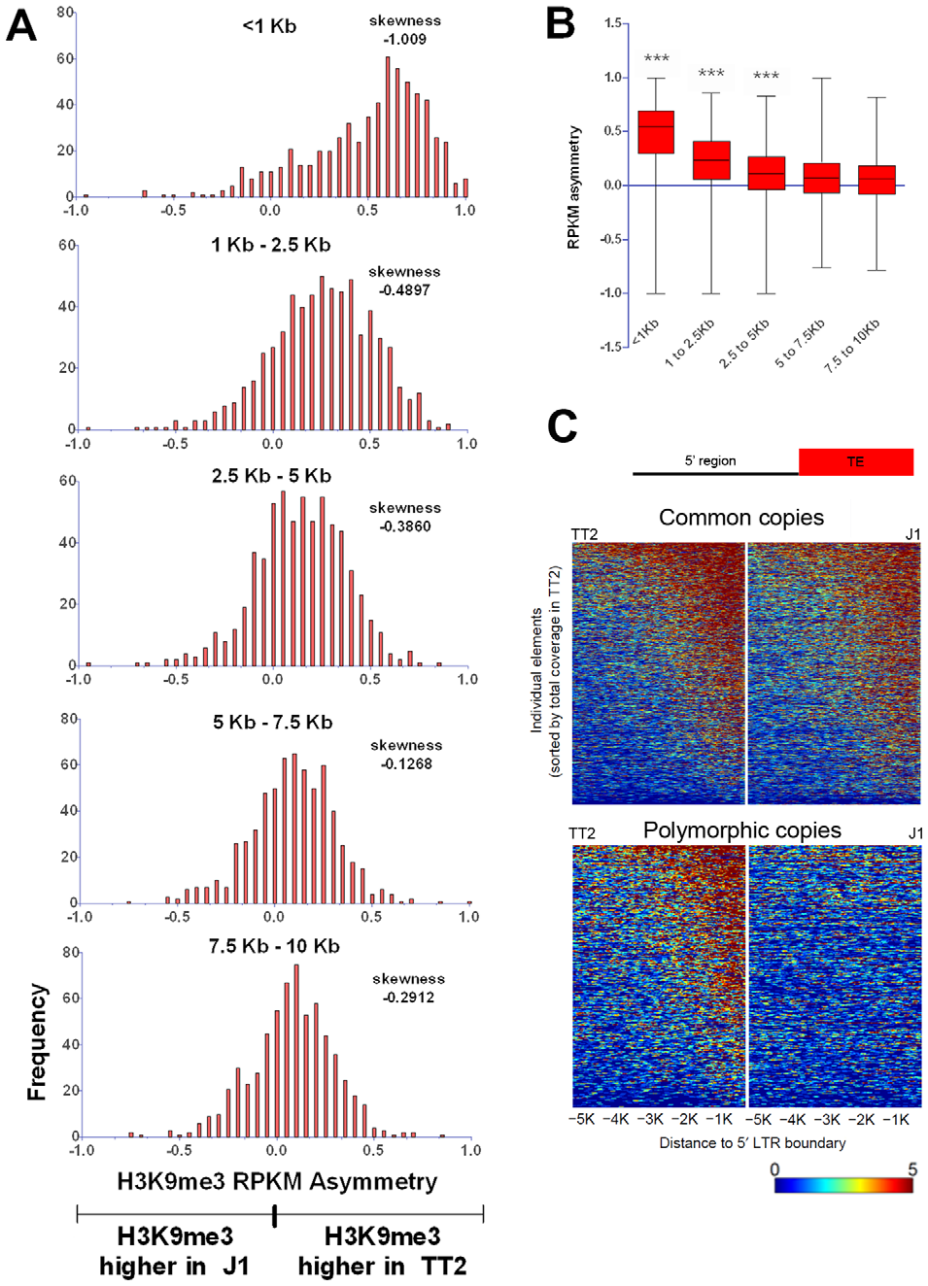
Length of polymorphic IAP-induced H3K9me3 chromatin. A. H3K9me3 RPKM asymmetry of insertionally polymorphic IAP copies was calculated for non-overlapping windows of 1.5 kb or 2.5 kb. The <1 kb window reflects the same data depicted in Figure 1C for IAP insertionally polymorphic copies (639 copies). Frequency is the number of copies having a given RPKM asymmetry. B. Whiskers on the sum of H3K9me3 asymmetry for all the different distances analyzed. Kruskal-Wallis and Dunn comparison tests were run and p values <0.001 are shown. C. Heatmap of H3K9me3 spreading in the 5′ flanking sequences of both common and polymorphic copies (for the 3′ region see Figure S12).

Despite such few documented examples, spreading of repressive chromatin is nevertheless often cited as a potential consequence of TE presence [50], [51]. From the insertionally polymorphic families that we analyzed, IAPs would likely be the only family of TEs capable of robustly spreading heterochromatin since they are able to consistently induce its formation in their proximal neighborhood. We measured the extent of IAP induced spreading of the H3K9me3 mark into flanking sequences by examining non-overlapping windows of 2.5 kb from the insertion sites of polymorphic copies (Figure 5A and 5B). Only the first one kb surrounding IAP copies is markedly affected by the IAP insertion. Even though the following 1.5 kb is still biased towards higher H3K9me3 associated with the IAP, the skewing is not as obvious as in the first one kb. However, it is important to note that some IAP copies are able to induce spreading of the H3K9me3 mark for almost 5 kb (Figure 5, Figure 1B and Figures S12 and S13). The analysis of other ERV families, discussed above, shows a similar degree of spreading for ERVK10C but spreading from members of the other ERV families appears more limited in extent (Figure 2). Therefore, only the closest regions seem to be robustly marked by H3K9me3 as a result of a nearby ERV and as observed above.

### Disruptive spreading into genes is rare

Given that H3K9me3 and H4K20me3, both of which have been shown to act as repressive marks, frequently extend at least one kb into genomic regions flanking IAP elements, we next determined whether such spreading could have a consequence on expression of neighboring host genes. We filtered the IAP polymorphic database for copies near transcription start sites (TSS – maximum distance of 5 kb) where H3K9me3 was detected at the IAP copy but also at the associated gene promoter. We required the gene promoter/TSS near the empty site not to be enriched in H3K9me3. Using RNA-seq data for both ES cell lines [16], we asked if the presence of a heterochromatic IAP copy might influence gene expression by comparing levels of RNA-seq reads in both lines. We found only one gene that matched these criteria. It is important to note, however, that IAPs near the TSS of genes are rare and therefore the number of IAP copies capable of disrupting gene expression is much lower than the total number of IAP copies analyzed. Indeed, from our dataset of polymorphic IAP copies [30], we found only four genes that harbor an upstream IAP copy less than 1 kb away (102 copies less than 5 kb away), and that differ in presence between strains C57BL/6 (TT2 cells) and 129 (J1 cells). Of these 102 cases, only one gene, *B3galtl*, a beta 1,3-galactosyltransferase-like gene, is differentially expressed in these two ES cell lines and this gene appears to be affected by heterochromatin spreading according to the criteria outlined above (see Figure S14 for genome browser view of this region).

**Figure 6:**
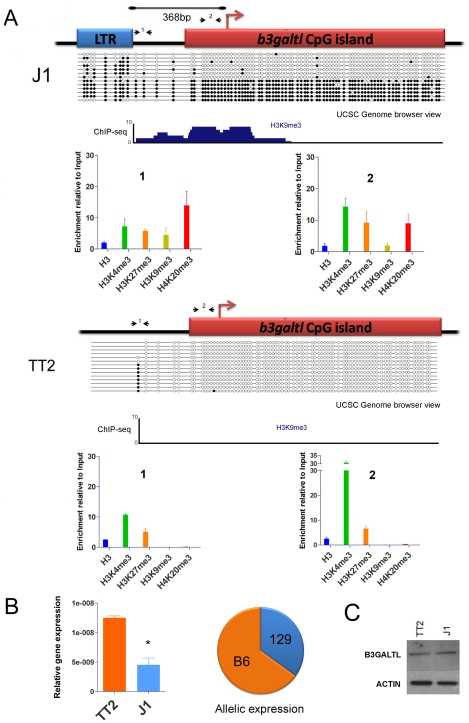
Impact of IAP-induced chromatin on the *B3galtl* gene. A. Full and empty site analysis of chromatin state and DNA methylation. Bisulfite methylation analysis and ChIP-qPCR were performed in TT2 (empty site) and J1 (full site) cell lines. The distance between the IAP copy and the transcriptional start site of the *B3galtl* gene in J1 is 368 bp. Small arrows represent qPCR primers used for both ES cell lines (fragments 1 and 2), dark circles are methylated CpGs and empty circles are unmethylated CpGs. The bisulfite representation diagram approximates the relative distance between CpGs. A genome browser view of the region analyzed by bisulfite is shown for H3K9me3 in both cell lines. Note that no enrichment of H3K9me3 is observed in TT2. A larger view of the *B3galtl* promoter and available histone marks is depicted in Figure S14. B. *B3galtl* expression in both ES cell lines and allelic expression in 129/B6 ES hybrids. RTqPCR was carried out in both cell lines, the mean of two biological replicates and standard deviation is depicted. The expression was calculated relative to the three most stable genes tested with genorm [75] (*actin*, *tubulin* and *TBP* – see Materials and Methods for more information). T student p-value = 0.0111. The pie chart represents the allelic expression in 129/B6 hybrid ES cells calculated as described in Materials and Methods. C. Western blot analysis of B3GALTL in TT2 (empty site) and J1 (full site) protein lysates. ACTIN is depicted to show no difference in protein loading. Westerns were done with biological triplicates and the results were consistent (Figure S16).

The *B3galtl* gene has a solitary antisense IAP LTR just 368bp upstream of the TSS in J1 ES cells (129 origin) but not in either allele of TT2 cells (C57BL/6 x CBA F1 hybrid) (Table 1). We studied chromatin post-translational modifications in the full site and empty site and in the CpG island promoter in both ES cell lines. At the full site only, we observe the appearance of more repressive marks, namely H3K9me3 and H4K20me3, associated with the presence of the IAP copy (Figure 6A and Figure S14). Furthermore, this gene has a CpG island promoter that is likely normally unmethylated, so we predict that spreading of DNA methylation into the CpG Island might accompany the repressive histone marks and have an important impact. The empty site (TT2) has no DNA methylation in the upstream region nor in the CpG Island (Figure 6A). There is significant H3K4me3 enrichment in the CpG Island in TT2 cells, as expected since this gene is expressed in these cells. On the contrary, the full site analysis (J1) shows that the IAP element itself is methylated in nearly all molecules sequenced. Strikingly, in a subset of these molecules, methylation spreads into the CpG Island. Since we are comparing two cell lines of different mouse strains, we wanted to ensure that the differences observed were indeed caused by the IAP and not by a different genetic background. Therefore we studied DNA methylation in B6/129 hybrid ES cells so both alleles are in the same background. Again, DNA was methylated in the CpG Island only in the full site (Figure S15). In both bisulfite analyses, the IAP copy was heavily methylated, however the spacer region between the IAP and the gene's CpG island can be methylated or unmethylated. Since the spacer region harbors different methylation patterns, we hypothesize that this region functions as a buffer that generally limits DNA methylation spreading from the IAP. Nevertheless, it appears that if the spacer region becomes methylated then the adjoining CpG Island will also be fully methylated.

Since we observe spreading of repressive marks into the CpG island of *B3Galtl,* we wondered if any expression differences could be observed between both strains as suggested by the RNA-seq data. Indeed, the presence of the IAP insertion is associated with a decrease in the RNA expression of *B3galtl* (Figure 6B). Allelic quantification in the hybrid cell line also shows a decrease in expression of the 129 allele (Figure 6B) suggesting that spreading of heterochromatin from the IAP copy is impeding expression from this allele. Nevertheless, no detectable difference in protein expression was observed between the cell lines (Figure 6C and Figure S16). Taken together, these observations reveal that an IAP element insertion near a gene can indeed induce local heterochromatin (DNA methylation, H3K9me3 and H4K20me3) and modify gene transcription. The lack of a significant difference in protein abundance suggests that posttranscriptional mechanisms compensate for the lower RNA levels in J1.

### Concluding remarks

**Figure 7:**
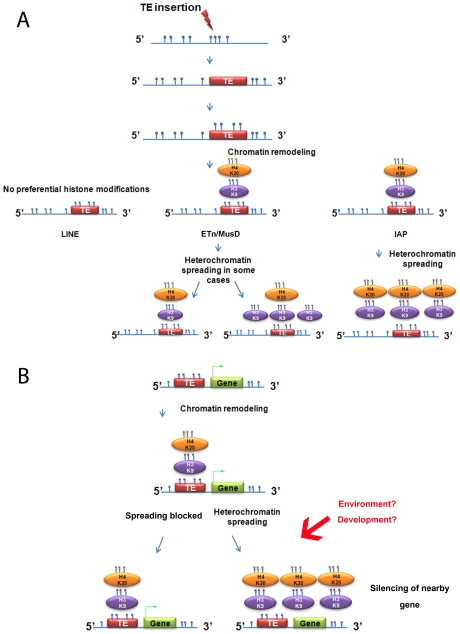
Summary of heterochromatin spreading due to transposable elements in mouse ES cell lines. A. Summary of the data obtained with the insertionally polymorphic families LINE, ETn/MusD and IAP. H4K20me3 on ETn/MusD was shown by Mikkelsen et al. [12]. B. Possible consequences of IAPs or ETn/MusDs near a gene. Blue circles represent methyl groups; if present directly on the DNA molecule they illustrate DNA methylation and if on an oval, a histone methylation. The green arrow on the gene represents transcription.

The strength of our model system to study induction of chromatin marks is the exploitation of natural insertional polymorphisms of TEs, which has advantages over an artificial system of introduced vectors that may not mimic natural loci. Using these polymorphic TEs, we demonstrate that, out of three abundant families of repeats in mouse, only IAPs consistently promote spreading of H3K9me3-H4K20me3-chromatin, robustly in the first 1 kb (Figure 7A). Indeed, the strong association of such chromatin and IAP copies allowed us to find new copies in the 129 mouse genome, but lacking in the sequenced B6 genome, by examining H3K9me3 regions differently enriched between both cell lines (Text S2). In contrast, LINE copies are not enriched in H3K9me3 and ETn/MusD are able to induce H3K9me3 chromatin only in some cases. The mechanisms and the nature of the IAP-induced heterochromatin are most likely responsible for the differences observed between IAPs, ETn/MusDs and LINEs as explained above. Indeed, the mechanisms regulating ERVs or LINEs are different in ES cells. IAPs and ETn/MusD are upregulated in mutants associated with H3K9me3 heterochromatin formation (ESET/Setdb1 [14]), the TRIM28/KAP1 pathway [15], and PcG complexes [41]) while LINEs are only modestly upregulated in such mutants [15] and mainly upregulated in DNA methylation mutants [7], [52]. DNA methylation influence on IAP copies in ES cells remains poorly understood as mutants of DNA methylation pathways (DNMT total KO) do not induce transcriptional upregulation of IAP copies while treatment with 5-azacytidine induces IAP over expression [15], [53]. Such a discrepancy could be explained by a recent report that the H3K9me3 genome-wide pattern in human cells is dramatically disturbed after treatment with 5-azacytidine [54]. As we observed, insertion sites for IAP copies are methylated at empty sites, suggesting that DNA methylation is not dependent on the presence of the IAP. Since the mouse genome is thought to be broadly methylated, one might extrapolate the results obtained for IAP empty sites to all TE empty sites. Henceforth, the global impact on the host genome of L1 copies probably involves other mechanisms than spreading of DNA methylation.

The analysis of other ERVs annotated in the sequenced genome shows that spreading of H3K9me3 towards flanking sequences is associated with regulation of the ERV family by this mark. However, the degree of deposition of this mark and also the spreading distance of such repressive chromatin seems to be unique for each of the families analyzed. There are two possible explanations for this observation. First, the degree of spreading of a TE family could be dependent on the number of copies that are actually targeted by H3K9me3, with copies marked with equivalent levels of H3K9me3 promoting equivalent spreading of heterochromatin. Alternatively, the degree of spreading of a TE family may be dependent on specific characteristics of each family, such that copies with equivalent H3K9me3 marking, if belonging to different families, would differently spread heterochromatin towards flanking regions. Our data cannot distinguish between these possibilities.

The mechanisms responsible for heterochromatin initiation in ERV copies in ES cells were recently studied. Small RNAs have been reported to act as central players in the formation and spreading of heterochromatin in several other species such as fission yeast and fruit flies, however in mammals such a role for small RNAs remains uncertain. It has been shown that DICER related pathways are not responsible for IAP repression [53]. Dicer-independent small RNAs, such as piRNAs, have only been described in the male germline of mouse [55]. Nevertheless, the influence of such small RNAs on the spreading of chromatin induced by IAPs and other ERVs should not be ruled out. Furthermore, we and others have shown that KAP1/Trim28 recruitment of SETDB1 is necessary for H3K9me3 silencing of ERVs in mouse ES cells [14], [15]. KAP1 along with KRAB-zinc finger proteins are able to induce spreading of repressive chromatin within 10 kb from the heterochromatin initiation site in humans [56] and such a mechanism might therefore be responsible for IAP induced-heterochromatin spreading in mouse.

Transposable elements have different life cycles and are expressed in different tissues and stages of development. It is well known that ETns are highly expressed in early development and then silenced [57]. Transgenic introduced IAPs are transcribed nearly exclusively in the male germ line [46] but expression of endogenous copies can be detected in thymocytes and other tissues [58]. Moreover, IAPs may become active in somatic tissues of old mice by demethylation of their sequences [45]. Mouse L1s have different expression patterns and even produce protein in testis for instance [59]. Therefore the time and place where the spreading of heterochromatin from these different families occurs may be different. Further analysis of other cell types, developmental stages and also other mouse strains would be of interest to compare to the results described here. Furthermore, epigenetic regulation of TEs may be influenced by environmental factors as already observed for several TEs [60]–[62]. Therefore, the induction and spreading of heterochromatin from a TE may be labile to environment and should be further studied in stressed conditions and during development. TEs may therefore provide cryptic sites for heterochromatin formation and also spreading.

We show that IAP induced repressive chromatin can affect the CpG island promoter of a neighboring mouse gene in cis, and in turn reduce expression of the genic mRNA. The paucity of examples of such a phenomenon is likely due to the fact that insertions of ERVs which attract and spread repressive chromatin and which occur very close to gene transcriptional start sites are strongly selected against unless such spreading is blocked (Figure 7B). Intriguingly, there are numerous situations where ERV LTRs are actually co-opted as constitutive, tissue-specific or developmental-specific promoters or enhancers for genes [63]–[65], indicating that the relationship between ERVs and genes is complex and multi-faceted. Even if the impact of ERV-induced heterochromatin is rare, it may participate in malleability of the host genome as epigenetic regulation of IAP copies and other TEs may be tissue or developmental-stage specific but also susceptible to environmental changes [66] (Figure 7B). The impact of TEs in genome evolution and speciation is being increasingly appreciated [2] and our report suggests that some TEs may have an indirect impact on host adaptive potential by spreading of epigenetic marks. As described for the sexual determination of melon [29], IAPs and other ERVs may have played a role in the genome evolution of *Mus musculus* through fine-tuning of genes by ERV-induced-heterochromatin. Indeed, since new insertions of IAPs continue to bombard the mouse genome, this fine-tuning of gene expression is likely ongoing and may contribute to phenotypic differences between strains.

## Materials and Methods

### Biological material

J1 and TT2 ES cells were passaged every 48–72 hours in DMEM supplemented with 15% FBS (HyClone), 20 mM HEPES, 0.1 mM nonessential amino acids, 0.1 mM 2-mercaptoethanol, 100 units/ml penicillin, 0.05 mM streptomycin, leukemia inhibitory factor (LIF) and 2mM glutamine on gelatinized plates.

### Transposable element copies

Common and polymorphic copies of ETn/MusD and IAP were obtained from our previous analysis of different strains of mouse [30]. In our analysis, ETn and IAP subtypes are grouped as a major family. Coordinates for regions containing LINE copies were obtained from [16] and include only the L1MdA subfamily. All coordinates depicted in figures and bioinformatics analyses refer to the sequenced mouse genome, July 2007 (NCBI37/mm9).

### ChIP-seq and MeDIP-seq

Methods and details on H3K9me3 ChIP-seq can be found in Karimi et al. [16]. For the layout of the experiments please see Figure S2.

MeDIP-seq libraries were constructed as described in Harris et al. [67], from 1 µg of genomic DNA using an anti-5-Methylcytidine monoclonal antibody obtained from Eurogentec (cat# BI-MECY-0100, lot#080808) and sequenced on an Illumina Genome Analyzer_iix_ following the manufacturer's recommended protocol (Illumina Inc., Hayward, CA). The resulting sequence reads were aligned using BWA v0.5.7 [68] using default parameters to the mouse reference genome (mm9). Uniquely placed sequence reads with a mapping quality of > = 10 were passed to FindPeaks v4.1 [69] for segmentation and wig [70] track generation with -dist_type = 0 [200], -duplicatefilter and no thresholding. After filtering, 23,293,703 and 23,672,774 reads remained for the TT2 and J1 libraries respectively. Custom Java program was used to calculate RPKM values for genomic regions of interest. RPKM was calculated using normalized genome coverage.

### Bioinformatics analyses

To compare total average density of H3K9me3 between two cell lines we used only full-length elements including flanking LTRs. For example out of 7,666 IAPE elements annotated in UCSC (mm9), only 1,318 (945 common and 373 polymorphic) satisfied the length selection criteria (total length > = 4000 bp) and were used in the analysis. Using the strand information, for every copy we identified the 5′ and 3′ 5 kb flanking regions and calculated coverage profiles in these regions for BWA aligned H3K9me3 reads directionally extended by 150 bp. To calculate total average density we agglomerated 5′ profiles and 3′ profiles for all elements for a given family and normalized them by 1) total number of copies in the family and 2) relative number of aligned reads in the library between TT2 and J1 cell lines. Reads were filtered by the BWA alignment quality (QC> = 7). If there were more than one sequenced read aligned to the same location, it was considered only once. Reads mapped to multiple locations were ignored.

In order to compare H3K9me3 or MeDIP coverage in specific regions between the two ES cell lines, a normalization factor has to be taken into account. We calculated the Reads per Kilobase per million mapped reads (denoted RPKM [71]) in all regions of interest for ChIP-seq samples. The following formula was used to calculate RPKM for these regions:
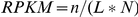



where *n* is a fractional number of reads aligned to the region, *L* is the length of the region in Kb, and *N* is the total number of aligned reads for a given sample, in millions.

For pair-wise H3K9me3 comparisons, we calculated the RPKM asymmetry across samples: 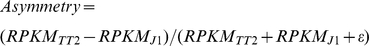



where RPKM_TT2_ and RPKM_J1_ are RPKMs in the region of interest of TT2 and J1 samples respectively, and 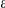 is a very small number to avoid dividing by zero. The asymmetry calculation gives us a number comprised between -1 (enriched only in J1) and +1 (enriched only in TT2) allowing us to directly compare H3K9me3 enrichment in specific regions between both cell lines. For further details on the ChIP-seq and RNA-seq data analysis refer to [16].

The skewness is a measure of the asymmetry of a distribution. If the skewness is equal to 0 the distribution is symmetric. When the skewness is negative, the left tail of the distribution is longer. When applied to our dataset it means that if the peak of frequency is >0 and the skewness is negative than the TT2 cells are more enriched than J1 at the loci analyzed. The skewness and other statistical tests (Kruskal-Wallis and Dunn comparison tests) were calculated using GraphPad Prism version 5.00 for Windows, GraphPad Software, San Diego California USA.

To plot heat maps of H3K9me3, we used the same coverage profiles as described above. Color indicates coverage at every base in the flank of individual copies. Rows corresponding to individual IAP elements were sorted according to the total coverage of H3K9me3 in the 5′ flank of the copies present in TT2 in the descending order. Same ordering of elements was preserved in the heat map showing H3K9me3 in J1.

To evaluate a mappability of a given genomic region we averaged genomic mappability (CRG Alignability) profiles that we downloaded in a form of bedGraph from the UCSC browser (http://genome.ucsc.edu [72]) and converted into a wig file. The value of mappability for every base in the genome for a given read length (50 bp in our case) gives a fraction of reads covering a given base and aligned uniquely to this genomic position even when up to two mismatches are allowed and ranges from 0 to 1.

### Chromatin immunoprecipitation and quantitative PCR

Two biological replicates of 10^7^ TT2 and J1 cells were used for Native ChIP. Cells were homogenized in a douncing buffer (10 mM Tris-HCl pH 7.5, 4 mM MgCl_2_, 1 mM CaCl_2_ and protease inhibitors) and disrupted using a p1000 tip and a syringe. Micrococcal nuclease digestion (150 U/ml) was performed for 7 min at 37°C and stopped with 10 mM EDTA. Correct digestion of the chromatin was verified by gel electrophoresis. Further lysis of the digested chromatin was done by incubating it 1 hour on ice with 1 ml of hypotonic lysis buffer (0.2 mM EDTA pH 8, 0.1 mM benzamidine, 0.1 mM PMSF, 1.5 mM DTT and protease inhibitors) and vortexing every 10 min. The digested chromatin was centrifuged at 3000g for 5 min and the supernatant was pre-cleared at 4°C for 2 hours in a rotating wheel with 100 µl of 50% solution of pre-blocked Protein A beads. Pre-cleared chromatin was aliquoted into 7 fractions (6 IP and 1 Input) and all IP fraction volumes were brought to 325 µl using IP Buffer (10 mM Tris-HCl, 1% Triton, 0.1% Deoxycholate, 0.1% SDS, 90 mM NaCl, 2 mM EDTA and protease inhibitors). We used antibodies recognizing total Histone H3 (Sigma, H9289), and histone modifications as H3K4me3 (Millipore, 17–614), H3K27me3 (Millipore, 07–449), H3K9me3 (Millipore, 07–442), H4K20me3 (Millipore, 17–671) and also IgG (12–370) as a negative control. After 1 hour at 4°C in a rotating wheel we added 20 µl of 50% solution of pre-blocked Protein A beads and rotated at 4°C overnight. Washes were held the next day by adding 400 µl of washing buffer to the beads (20 mM Tris-HCl pH 8, 0.1% SDS, 1% Triton X-100, 2 mM EDTA, 150 mM NaCl and protease inhibitors), rotating the samples for 3 min at 4°C and spinning at 4000 rpm. The amount of NaCl was increased in the second wash step (500 mM NaCl) and performed as described above. The INPUT and IP fractions were eluted with 100 µl of 100 mM NaHCO_3_ - 1% SDS. RNase treatment was performed for 2 hours at 68°C with gentle vortexing. The IP samples were spun at 4000 rpm for 2 min and the supernatant recovered (twice). All samples were purified with the Qiaquick PCR purification kit (Qiagen) and quantified by using the PicoGreen system from Invitrogen. 0.05 ng/µl of ChIP material was analyzed in technical duplicates through quantitative PCR (Fast SYBR Green Master Mix from Applied Biosystems) by comparing the amplification of Input DNA relative to immunoprecipitated DNA (IP) using the formula “Efficiency of primers∧(Ct^Input^ – Ct^IP^)” where the efficiency is calculated through serial dilutions of Input DNA (primers efficiency were all comprised between 1.9 and 2.1). ChIP enrichment for each specific antibody was tested with control regions for each antibody used for both ES cells (Figure S10). For H4K20me3, primers located more than one nucleosome away (150 bp) were tested to confirm spreading of this mark (Figure S9). All primers are listed in Table S1.

### Bisulfite conversion

Bisulfite conversion, PCR, cloning and sequencing were carried out as described previously [73]. All the sequences included in the analyses either displayed unique methylation patterns or unique C to T non-conversion errors (remaining C's not belonging to a CpG dinucleotide) after bisulfite treatment of the genomic DNA. This avoids considering several PCR-amplified sequences resulting from the same template molecule (provided by a single cell). All sequences had a conversion rate >95%. Sequences were analyzed with the Quma free online software [74].

### Expression analysis

Total RNA was extracted (two biological replicates for each ES cell line) with the All Prep DNA/RNA mini kit from Qiagen. RNA was treated with the Turbo DNA-free kit from Ambion in order to remove DNA. One µg of total RNA extracts was reverse transcribed with SuperScript II reverse transcriptase system (Invitrogen). We synthesized two different cDNAs (65°C for 5 min, 25°C for 5 min, 50°C for 60 min and 70°C for 15 min): a control reaction with no reverse transcriptase to test DNA contamination, and a pool of total cDNA synthesized with random primers. The cDNA samples were diluted 10 fold, and PCR was carried out using Fast SYBR Green Master Mix (Applied Biosystems) using specific primers for each gene analyzed. Primers were chosen surrounding introns in order to amplify 100-250 bp fragments of cDNA. Quantitative PCR cycling conditions were 20 s at 95°C (1 cycle), and then 3 s at 95°C, followed by 30 s at 60°C (45cycles). Reactions were done in duplicate, and standard curves were calculated from serial dilutions of cDNA. The quantity of the transcripts was estimated relative to the expression of *tubulin*, *actin* and *TBP* (TATA binding protein); chosen as the most stable genes out of 6 reference genes tested using the GeNorm method [75]; with the equation “Absolute quantity  =  “Efficiency of primers∧(-Ct)”. Primers efficiency were equivalent and chosen between 1.9 and 2. All primers are listed in Table S1.

For determining allele-specific expression, RNA extraction and cDNA synthesis was carried as described above. Allelic expression was determined with the PeakPicker software [76] based on chromatogram analysis of cDNA and genomic PCR fragments.

### Western blot

Western blot was done with biological triplicates. Cells were washed in PBS and solubilised in RIPA buffer (150 mM NaCl, Triton-X 1%, 50 mM Tris-HCl pH8, 0.1% SDS and 50 mM sodium deoxycholate) for 20 min at 4°C. For performing Western blot analysis, total cell extracts (40 µg/lane) were size-fractionated by NuPAGE 4-12% Bis-Tris Gel (Invitrogen) and transferred to polyvinylidene difluoride membranes. The membranes were blocked for one hour and then incubated with first antibodies against B3GALTL (Santa Cruz sc-67610) and ACTIN (Sigma A2066) overnight. After washing, blots were incubated with the secondary antibodies for one hour (1:8000 Peroxidase rabbit anti-goat (Sigma A5420) and 1∶10000 goat anti-rabbit (Sigma A0545), and specifically bound antibodies were detected with the ECL (Thermo Scientific). The films were then scanned and the quantification of B3GALTL protein was performed using Adobe Photoshop (San Jose, CA) with three biological replicates as previously described [77]. For each lane, the B3GALTL level was normalized to that of ACTIN (Figure S16).

## Supporting Information

Figure S1 Distribution of common and polymorphic copies of IAP and ETn/MusD families and of repeat masker annotated L1Md copies. A. Ensembl view of all common and polymorphic copies. Each arrow represents an insertion, but since the number of TE copies is high, some loci show superposed arrows. B. Distance to genes for IAPs and ETn/MusD. The distance was grouped as in Figure 5.
(PDF)

Figure S2 Chromatin immunoprecipitation and sequencing chart protocol.
(TIF)

Figure S3 A. RNA-seq and H3K9me3-seq enrichment per copy on ERV and LINE families studied. Asterisks denote the qualitative strength of H3K9me3 spreading as in Figure 2. B. Single copy analysis of expression and H3K9me3 coverage. Uniquely aligned reads were used for single copy expression and H3K9me3 coverage of the flanking regions only is plotted. Note that using uniquely mapped reads results in a bias towards old copies that have accumulated diagnostic mutations and are very often silenced or unable to produce any transcript. We are therefore underestimating the number of expressed copies. C. Single copy H3K9me3 mappability for common and polymorphic IAP and ETn/MusD elements and their flanking regions. A score of one denotes 100% mappability, and 0, 0%.
(PDF)

Figure S4 H3K9me3 RPKM of flanking regions for J1 and TT2 cell lines. Black lines represent equal distribution of J1 and TT2 H3K9me3 RPKM (y = x).
(PDF)

Figure S5 RPKM asymmetry of three random sets of 69 IAP copies. Skewing towards higher H3K9me3 in full sites is still observed with IAP copies even when fewer copies are analyzed.
(PDF)

Figure S6 H3K9me3 RPKM asymmetry for full-length and solo-LTR ETn/MusD copies. Full-length copies were chosen empirically larger than 4 Kb. Solo-LTRs were chosen as being ±20% of a typical solo-LTR size (320 bp). The percentage of LTR identity was calculated by retrieving the LTR sequences with a typical LTR probe and aligning both LTRs of the same full-length copy with blast2 [78]. The data set is comprised of 249 common full-length copies, 719 common solo LTRs, 6 insertionally polymorphic MusDs, 22 insertionally polymorphic full-length ETns and 32 insertionally polymorphic solo LTRs.
(PDF)

Figure S7 Genome browser view of the five IAP copies chosen to characterize IAP-induced heterochromatin.
(PDF)

Figure S8 H3K9me3 RPKM and ChIP for the five copies analyzed in both ES cell lines. TT2 Left and TT2 Right correspond to the upstream and downstream adjacent regions to the IAP insertion.
(PDF)

Figure S9 H4K20me3 enrichment of the five IAP copies studied in Figure 3.
(TIF)

Figure S10 ChIP-qPCR positive and negative controls. H4K20me3 region was chosen with the Mikkelsen et al. data set [12].
(PDF)

Figure S11 Bisulfite sequencing of full and empty sites of five IAP copies. Statistical analysis of methylation of flanking regions is shown. A star illustrates a significant increase in DNA methylation in the region analyzed.
(PDF)

Figure S12 Heatmap of H3K9me3 spreading in the 3′ flanking sequences of both common and polymorphic copies (for the 5′ region see Figure 5C).
(PDF)

Figure S13 Genome Browser view of IAP copies showing H3K9me3 spreading for several kb.
(PDF)

Figure S14 UCSC Genome Browser view of *B3galtl*. The polymorphic ERV track shows a black bar for the IAP insertion in the 129 (J1) genome. H3K9me3 ChIP-seq data represented is from Karimi et al. [16], obtained from TT2 and J1 ES cells. H3K4me3, H3K27me3 and H4K20me3 data was obtained from the study of Mikkelsen et al. [12] and is shown for comparison but note this data was generated from hybrid ES cells (129SvJae x *M. castaneus* F1; male). The IAP insertion is present in the 129 allele but is absent from the *M. castaneus* allele. Hence, enrichment observed for histone marks obtained in these hybrid ES cells should be viewed with caution.
(PDF)

Figure S15 Bisulfite analysis of DNA from hybrid B6/129 ES cells of the *B3galtl* locus.
(PDF)

Figure S16 Western blot on three biological replicates of TT2 and J1 cell lines with anti- B3GALTL and -ACTIN.
(PDF)

Table S1 Primers for ChIP-qPCR and RT-qPCR.
(PDF)

Text S1 ETn/MusD elements are variably regulated by epigenetic mechanisms. References cited are [79], [80].
(PDF)

Text S2 New putative IAP copies in ES cell lines. References cited are [81], [82].
(PDF)
