# Supplementary figures and images for: Retrotransposon-Induced Heterochromatin Spreading in the Mouse Revealed by Insertional Polymorphisms
Source: PLoS Genet. 2011 Sep 29;7(9):e1002301. doi: 10.1371/journal.pgen.1002301 (PMC3183085; doi:10.1371/journal.pgen.1002301)

A

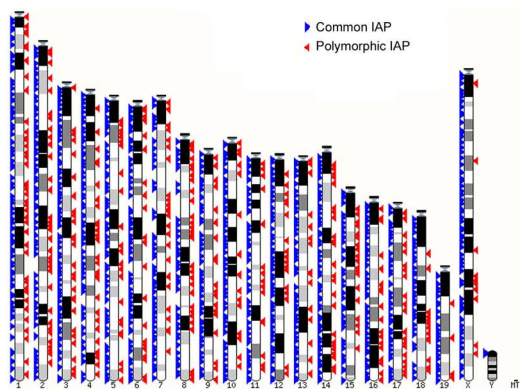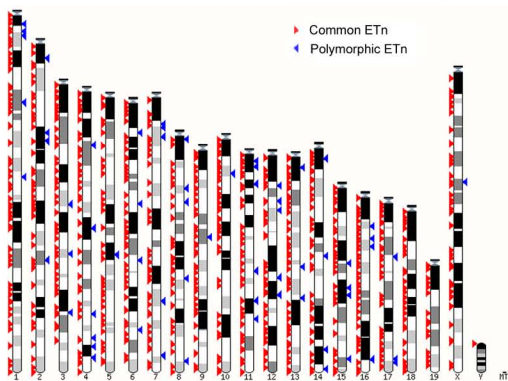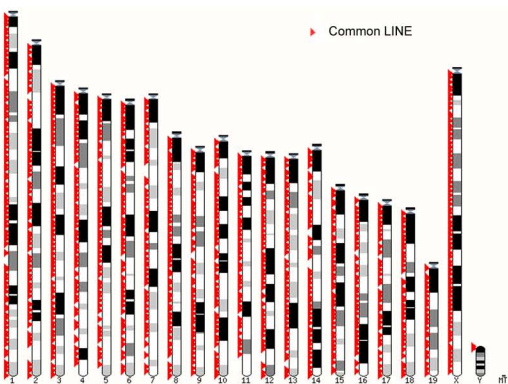

B

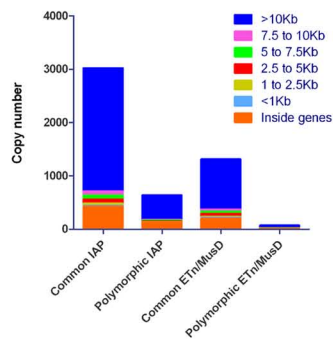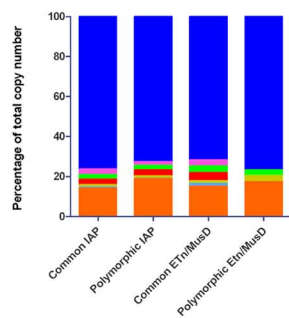

Supplement: Figure S1 — Distribution of common and polymorphic copies of IAP and ETn/MusD families and of repeat masker annotated L1Md copies. A. Ensembl view of all common and polymorphic copies. Each arrow represents an insertion, but since the number of TE copies is high, some loci show superposed arrows. B. Distance to genes for IAPs and ETn/MusD. The distance was grouped as in Figure 5. (PDF) [file pgen.1002301.s001.pdf]

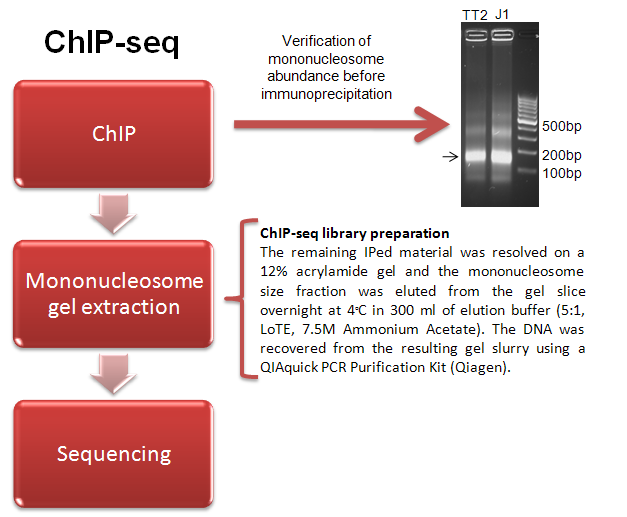

Supplement: Figure S2 — Chromatin immunoprecipitation and sequencing chart protocol. (TIF) [file pgen.1002301.s002.tif]

A

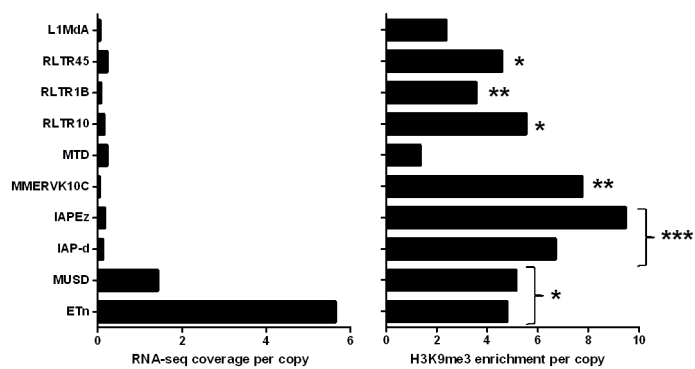

B

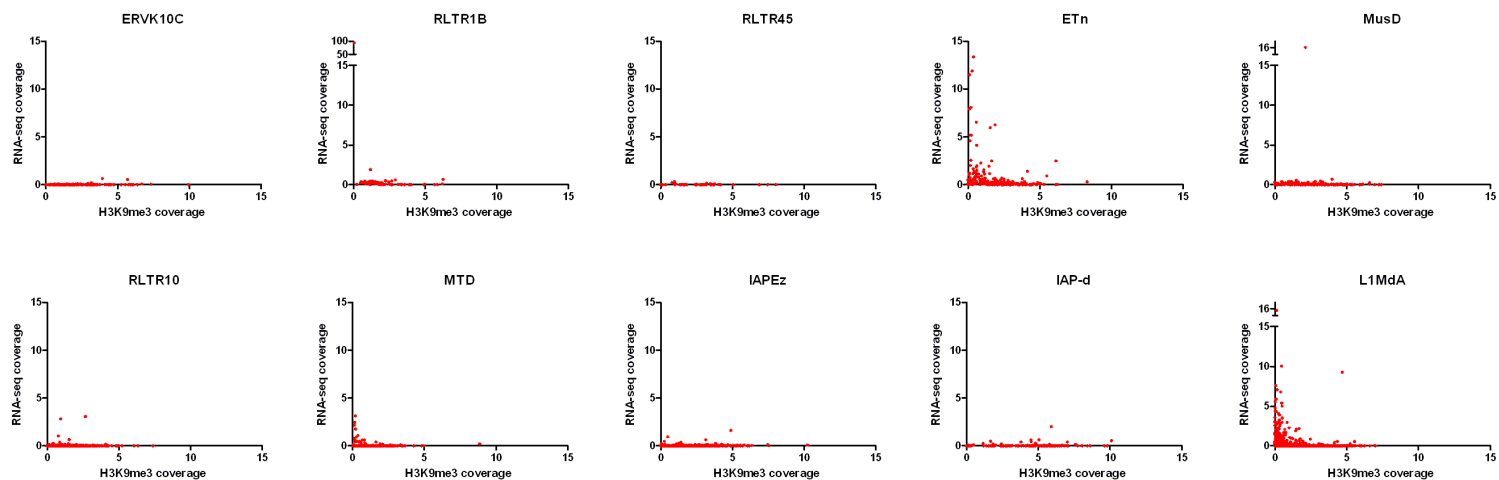

C

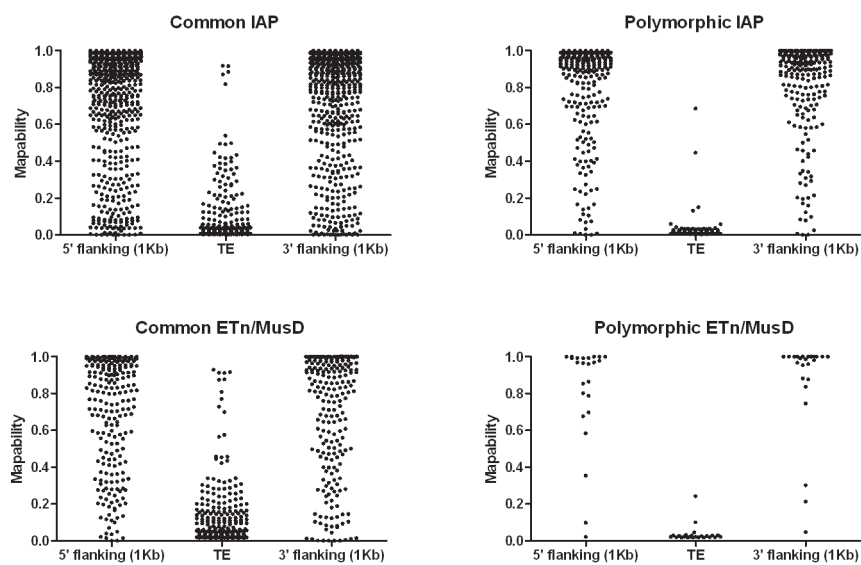

Supplement: Figure S3 — A. RNA-seq and H3K9me3-seq enrichment per copy on ERV and LINE families studied. Asterisks denote the qualitative strength of H3K9me3 spreading as in Figure 2. B. Single copy analysis of expression and H3K9me3 coverage. Uniquely aligned reads were used for single copy expression and H3K9me3 coverage of the flanking regions only is plotted. Note that using uniquely mapped reads results in a bias towards old copies that have accumulated diagnostic mutations and are very often silenced or unable to produce any transcript. We are therefore underestimating the number of expressed copies. C. Single copy H3K9me3 mappability for common and polymorphic IAP and ETn/MusD elements and their flanking regions. A score of one denotes 100% mappability, and 0, 0%. (PDF) [file pgen.1002301.s003.pdf]

**Common ETn/MusDs**

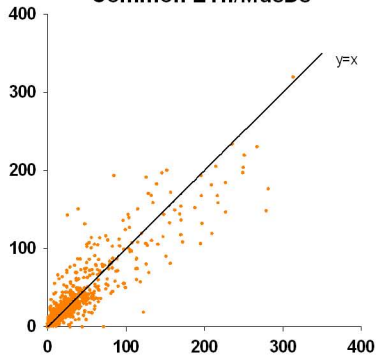

**Common IAPs**

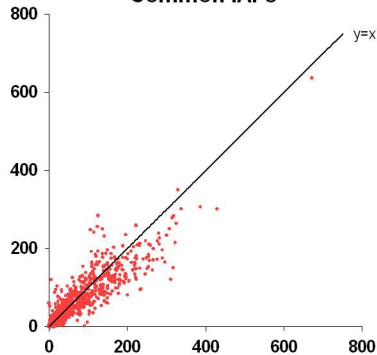

**Polymorphic ETn/MusDs**

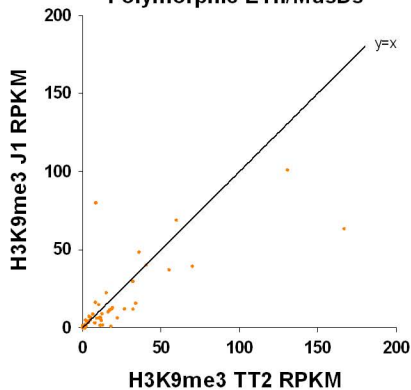

**Polymorphic IAPs**

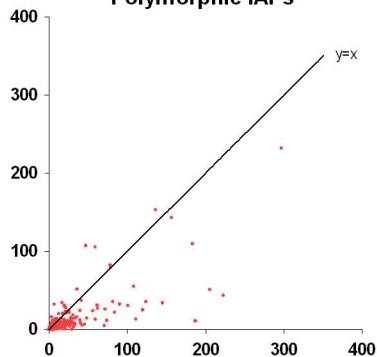

Supplement: Figure S4 — H3K9me3 RPKM of flanking regions for J1 and TT2 cell lines. Black lines represent equal distribution of J1 and TT2 H3K9me3 RPKM (y = x). (PDF) [file pgen.1002301.s004.pdf]

## Common IAP

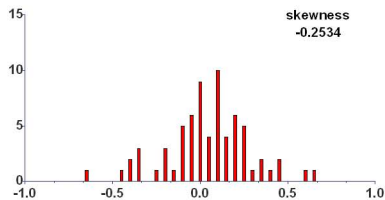

## Polymorphic IAP

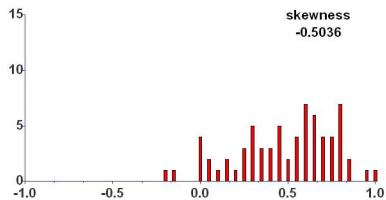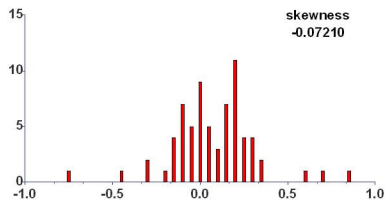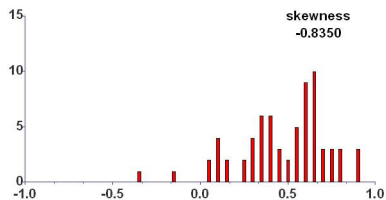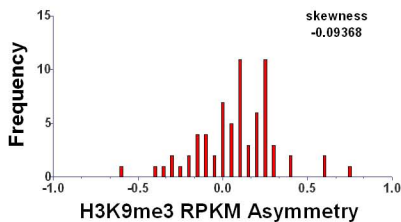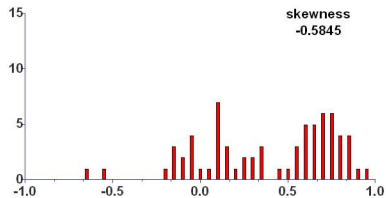

H3K9me3 higher in J1      H3K9me3 higher in TT2

Random set of copies

Supplement: Figure S5 — RPKM asymmetry of three random sets of 69 IAP copies. Skewing towards higher H3K9me3 in full sites is still observed with IAP copies even when fewer copies are analyzed. (PDF) [file pgen.1002301.s005.pdf]

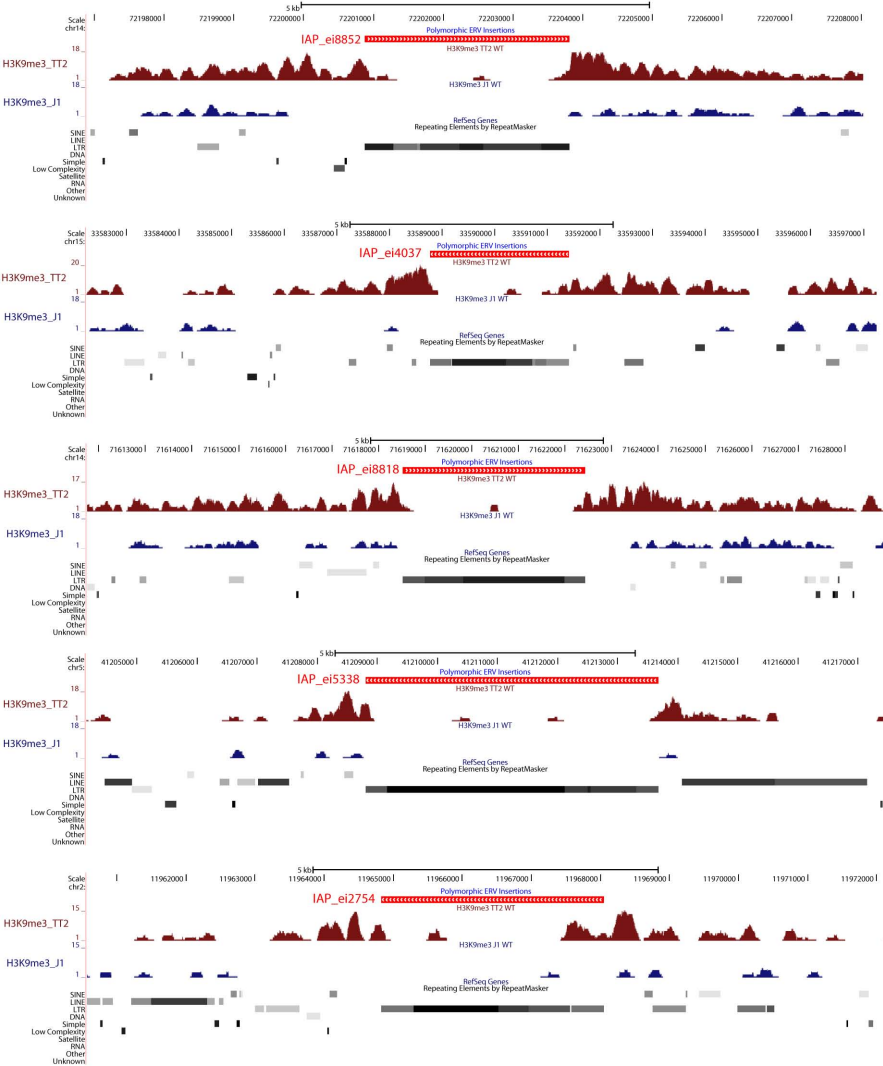

Supplement: Figure S7 — Genome browser view of the five IAP copies chosen to characterize IAP-induced heterochromatin. (PDF) [file pgen.1002301.s007.pdf]

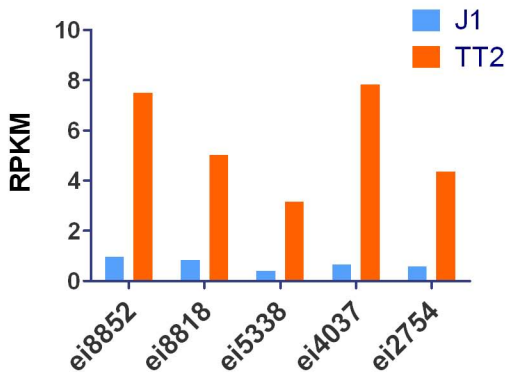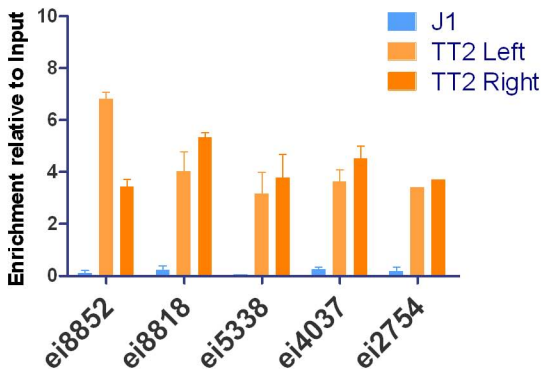

Supplement: Figure S8 — H3K9me3 RPKM and ChIP for the five copies analyzed in both ES cell lines. TT2 Left and TT2 Right correspond to the upstream and downstream adjacent regions to the IAP insertion. (PDF) [file pgen.1002301.s008.pdf]

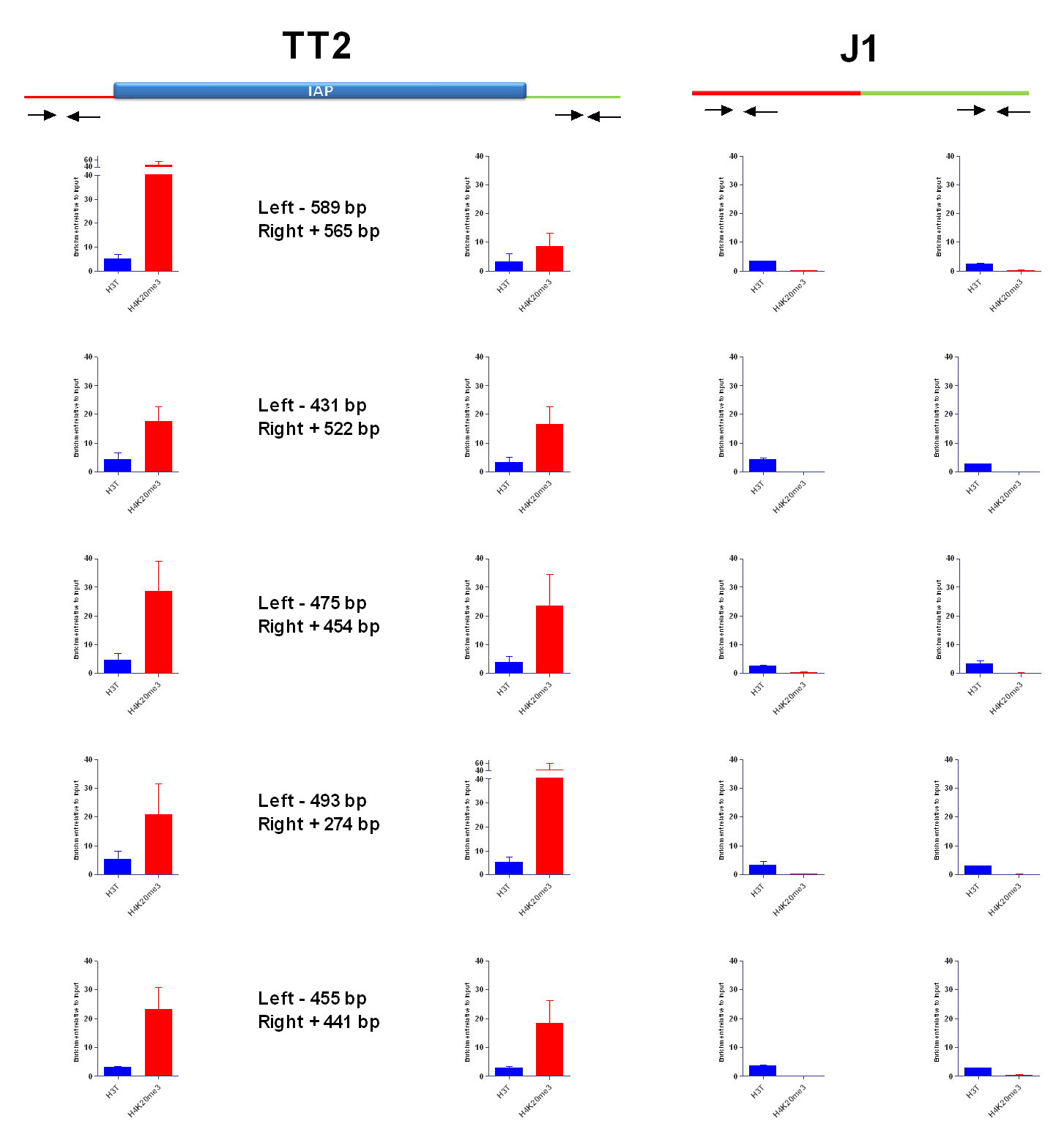

Supplement: Figure S9 — H4K20me3 enrichment of the five IAP copies studied in Figure 3. (TIF) [file pgen.1002301.s009.tif]

# Full site

# Empty site

ei8852

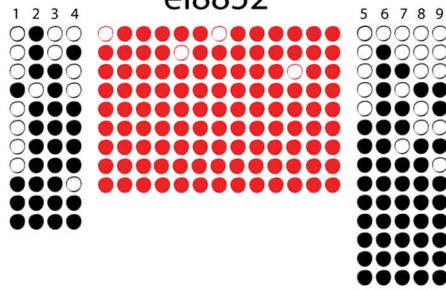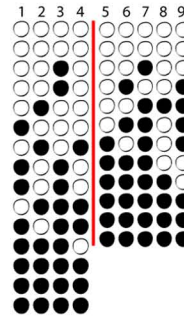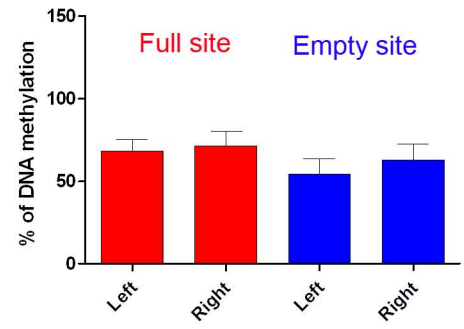

ei4037

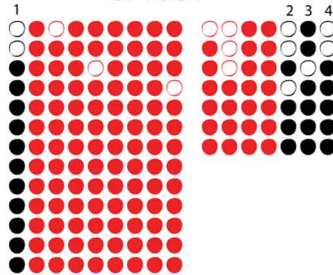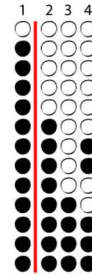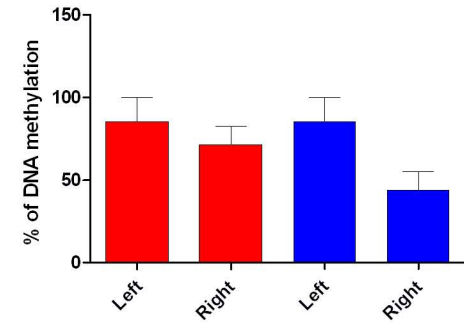

ei8818

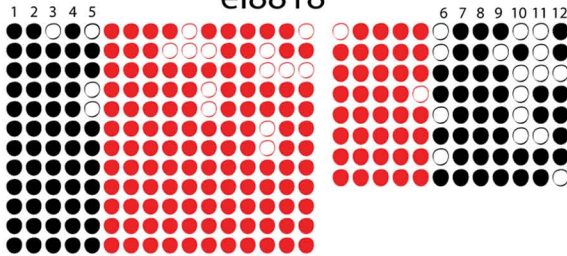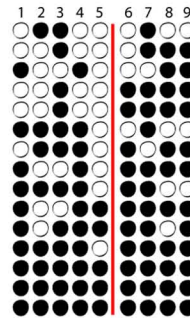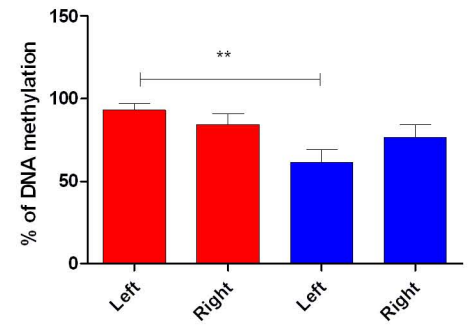

ei5338

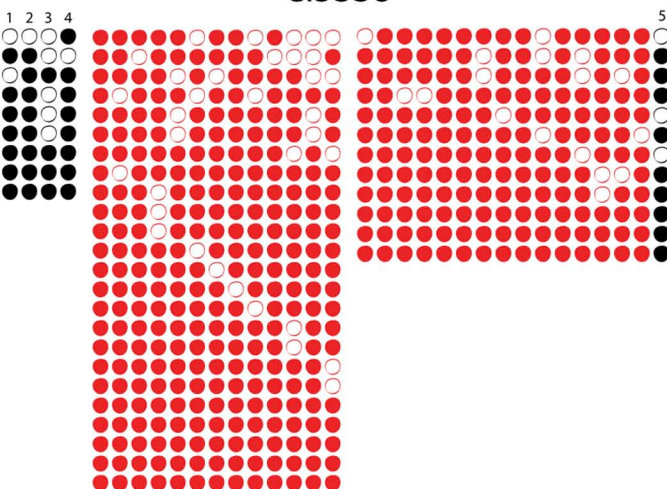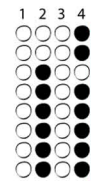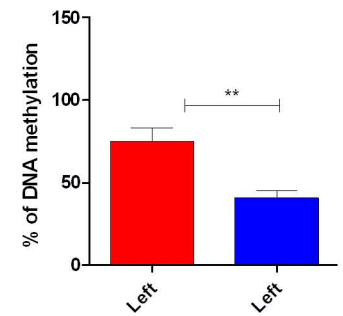

ei2754

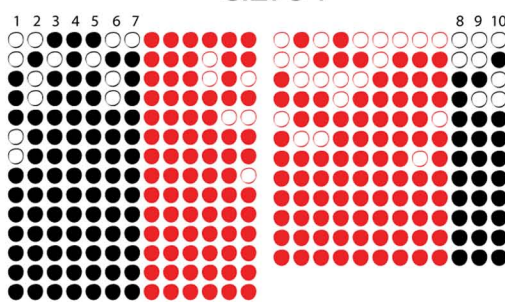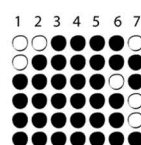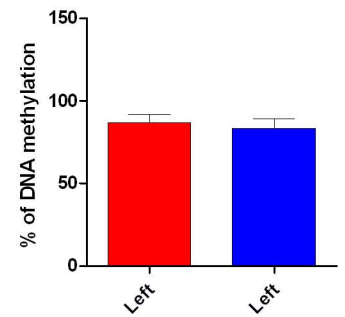

Supplement: Figure S11 — Bisulfite sequencing of full and empty sites of five IAP copies. Statistical analysis of methylation of flanking regions is shown. A star illustrates a significant increase in DNA methylation in the region analyzed. (PDF) [file pgen.1002301.s011.pdf]

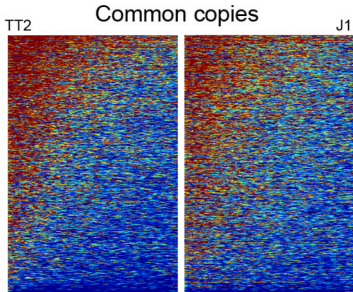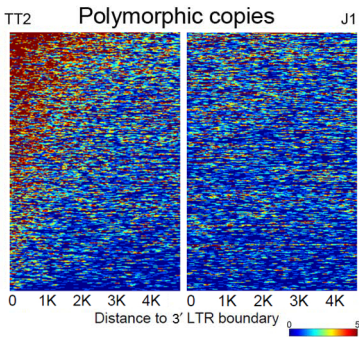

Supplement: Figure S12 — Heatmap of H3K9me3 spreading in the 3′ flanking sequences of both common and polymorphic copies (for the 5′ region see Figure 5C). (PDF) [file pgen.1002301.s012.pdf]

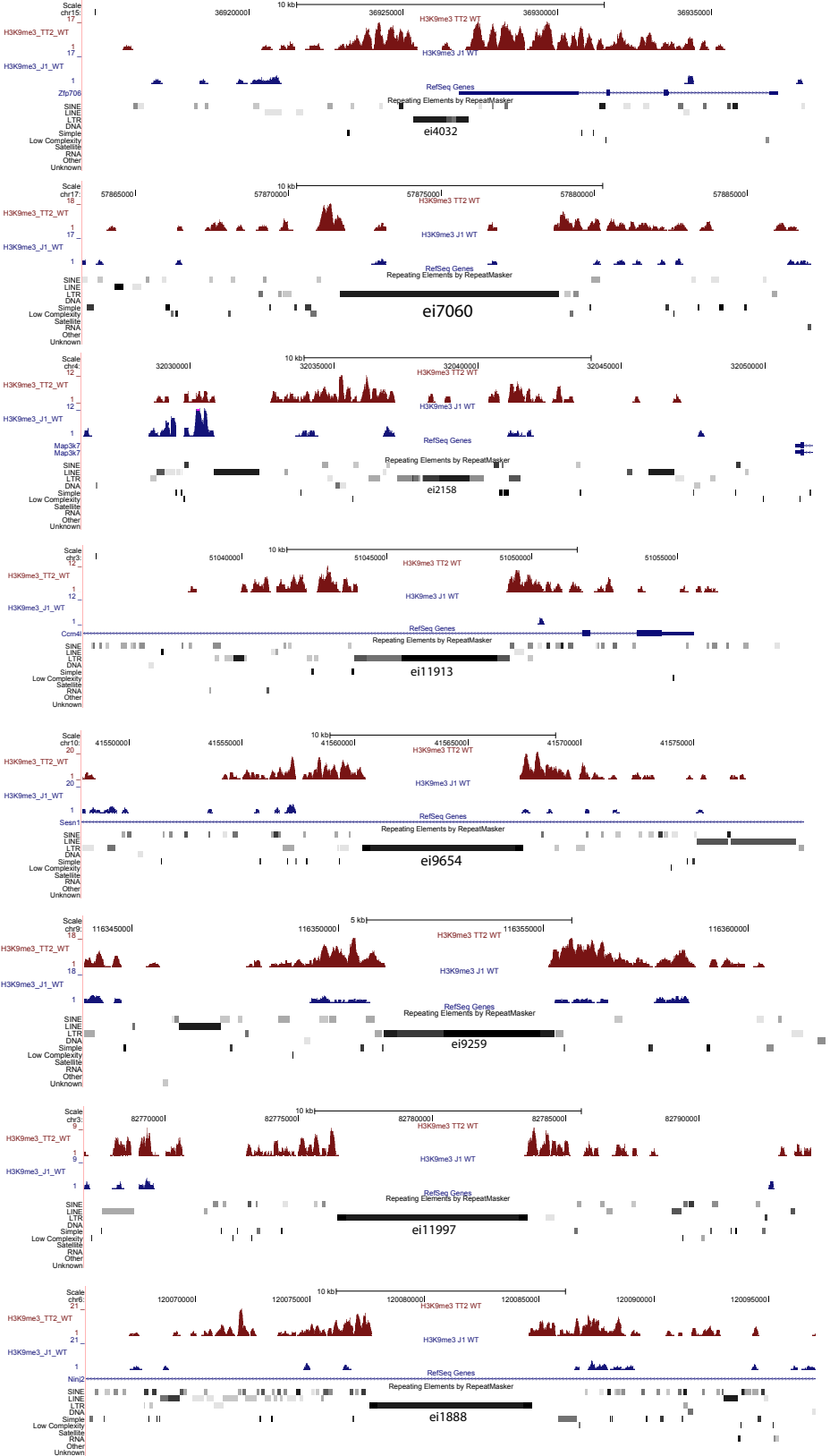

Supplement: Figure S13 — Genome Browser view of IAP copies showing H3K9me3 spreading for several kb. (PDF) [file pgen.1002301.s013.pdf]

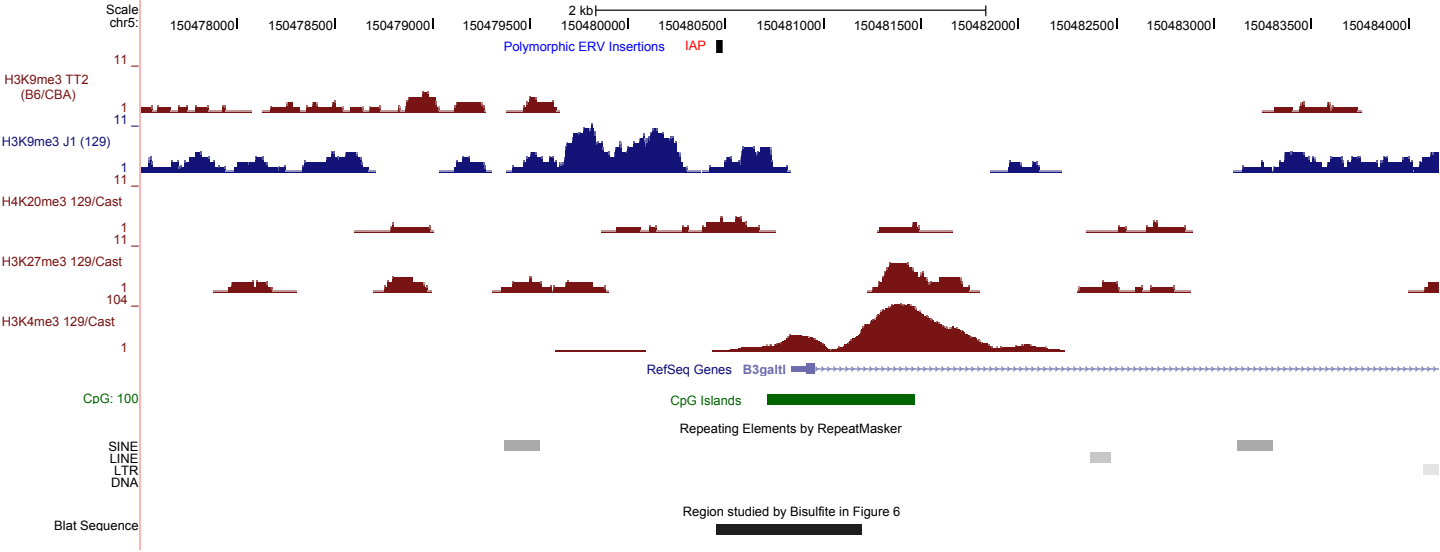

Supplement: Figure S14 — UCSC Genome Browser view of B3galtl. The polymorphic ERV track shows a black bar for the IAP insertion in the 129 (J1) genome. H3K9me3 ChIP-seq data represented is from Karimi et al. [16], obtained from TT2 and J1 ES cells. H3K4me3, H3K27me3 and H4K20me3 data was obtained from the study of Mikkelsen et al. [12] and is shown for comparison but note this data was generated from hybrid ES cells (129SvJae x M. castaneus F1; male). The IAP insertion is present in the 129 allele but is absent from the M. castaneus allele. Hence, enrichment observed for histone marks obtained in these hybrid ES cells should be viewed with caution. (PDF) [file pgen.1002301.s014.pdf]

129 allele

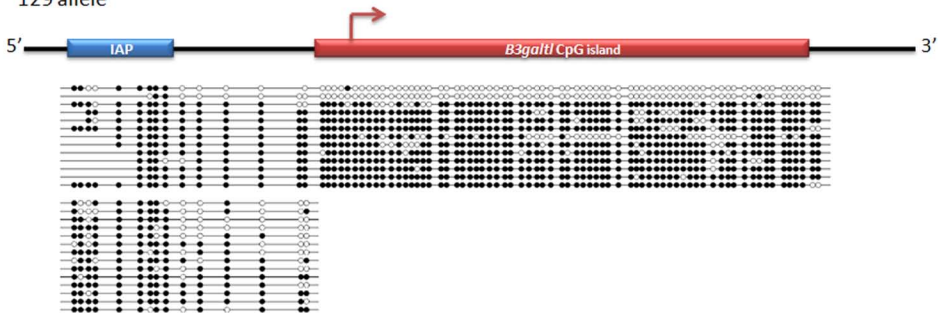

B6 allele

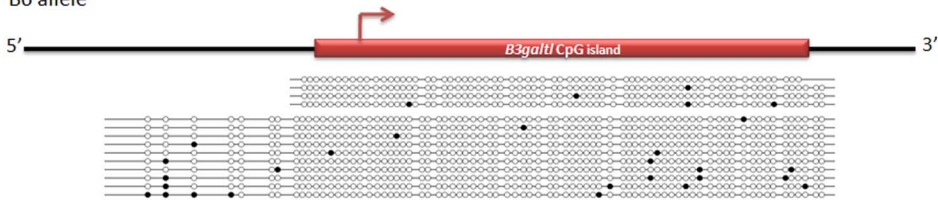

Supplement: Figure S15 — Bisulfite analysis of DNA from hybrid B6/129 ES cells of the B3galtl locus. (PDF) [file pgen.1002301.s015.pdf]

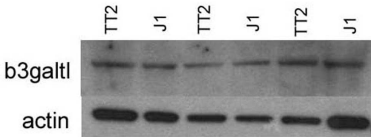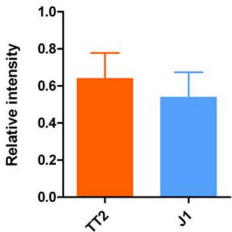

Supplement: Figure S16 — Western blot on three biological replicates of TT2 and J1 cell lines with anti- B3GALTL and -ACTIN. (PDF) [file pgen.1002301.s016.pdf]
