# Supplementary material for: Retrotransposon-Induced Heterochromatin Spreading in the Mouse Revealed by Insertional Polymorphisms
Source: PLoS Genet. 2011 Sep 29;7(9):e1002301. doi: 10.1371/journal.pgen.1002301 (PMC3183085; doi:10.1371/journal.pgen.1002301)
Supplement: Figure S6 — H3K9me3 RPKM asymmetry for full-length and solo-LTR ETn/MusD copies. Full-length copies were chosen empirically larger than 4 Kb. Solo-LTRs were chosen as being ±20% of a typical solo-LTR size (320 bp). The percentage of LTR identity was calculated by retrieving the LTR sequences with a typical LTR probe and aligning both LTRs of the same full-length copy with blast2 [78]. The data set is comprised of 249 common full-length copies, 719 common solo LTRs, 6 insertionally polymorphic MusDs, 22 insertionally polymorphic full-length ETns and 32 insertionally polymorphic solo LTRs. (PDF) [file pgen.1002301.s006.pdf]

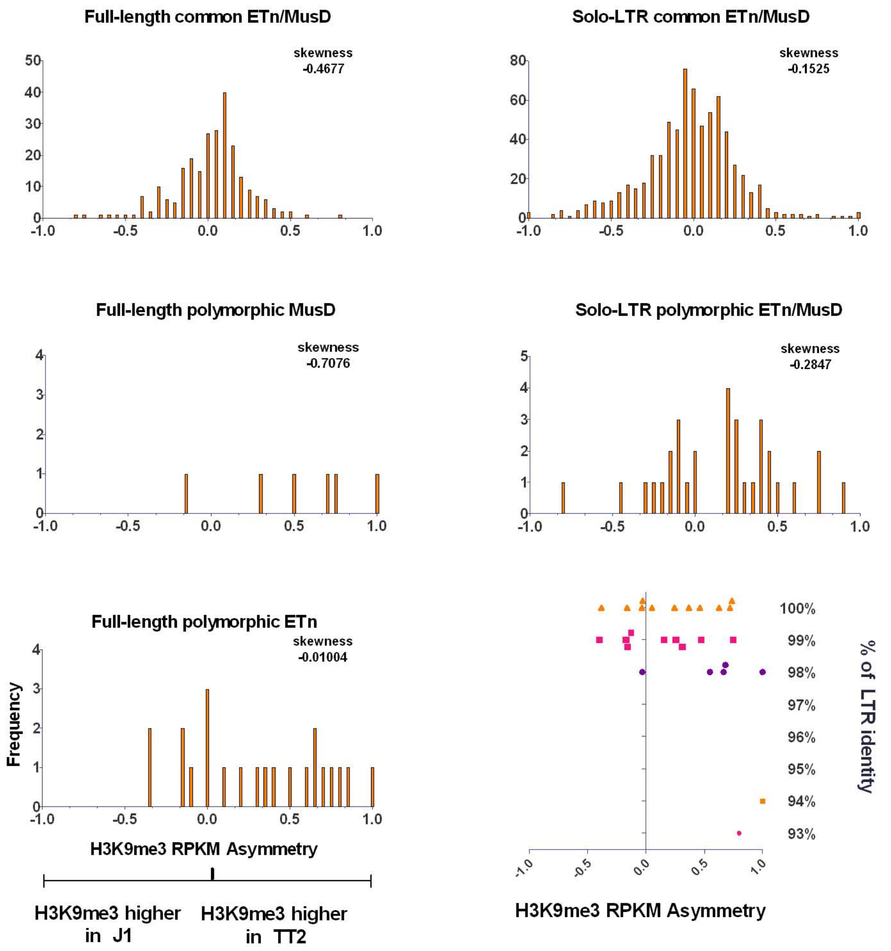

Full-length common ETn/MusD
skewness
-0.4677
Solo-LTR common ETn/MusD
skewness
-0.1525
Full-length polymorphic MusD
skewness
-0.7076
Solo-LTR polymorphic ETn/MusD
skewness
-0.2847
Full-length polymorphic ETn
skewness
-0.01004
Frequency
-1.0
-0.5
0.0
0.5
1.0
H3K9me3 RPKM Asymmetry
H3K9me3 higher in J1
H3K9me3 higher in TT2
100%
99%
98%
97%
96%
95%
94%
93%
% of LTR identity
H3K9me3 RPKM Asymmetry
