# Supplementary material for: Retrotransposon-Induced Heterochromatin Spreading in the Mouse Revealed by Insertional Polymorphisms
Source: PLoS Genet. 2011 Sep 29;7(9):e1002301. doi: 10.1371/journal.pgen.1002301 (PMC3183085; doi:10.1371/journal.pgen.1002301)
Supplement: Figure S10 — ChIP-qPCR positive and negative controls. H4K20me3 region was chosen with the Mikkelsen et al. data set [12]. (PDF) [file pgen.1002301.s010.pdf]

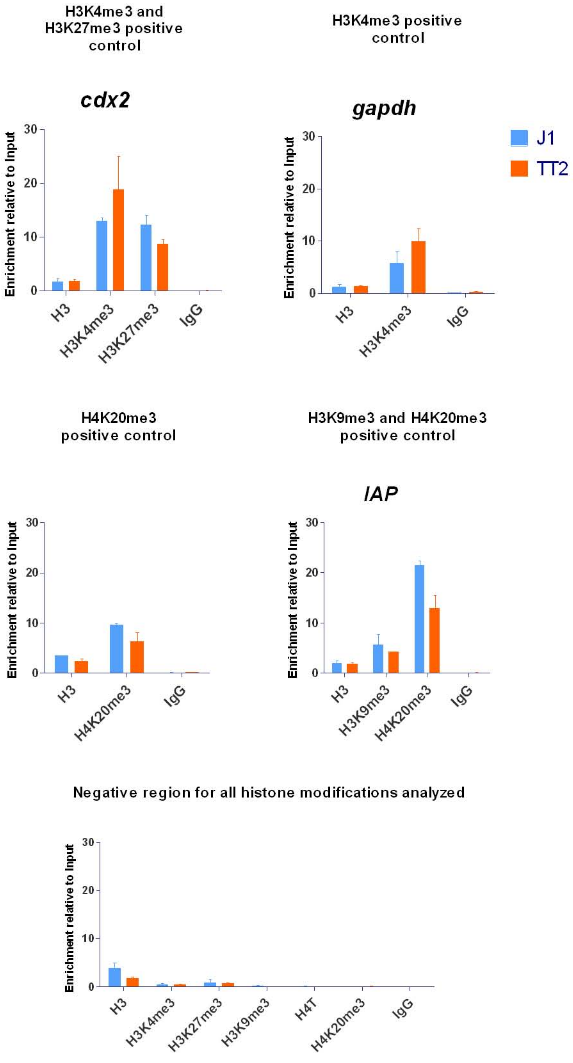

H3K4me3 and H3K27me3 positive control
cdx2
Enrichment relative to Input
30
20
10
0
H3
H3K4me3
H3K27me3
IgG
H3K4me3 positive control
gapdh
Enrichment relative to Input
30
20
10
0
H3
H3K4me3
IgG
J1
TT2
H4K20me3 positive control
Enrichment relative to Input
30
20
10
0
H3
H4K20me3
IgG
H3K9me3 and H4K20me3 positive control
IAP
Enrichment relative to Input
30
20
10
0
H3
H3K9me3
H4K20me3
IgG
Negative region for all histone modifications analyzed
Enrichment relative to Input
30
20
10
0
H3
H3K4me3
H3K27me3
H3K9me3
H4T
H4K20me3
IgG
