# Supplementary material for: Retrotransposon-Induced Heterochromatin Spreading in the Mouse Revealed by Insertional Polymorphisms
Source: PLoS Genet. 2011 Sep 29;7(9):e1002301. doi: 10.1371/journal.pgen.1002301 (PMC3183085; doi:10.1371/journal.pgen.1002301)
Supplement: Table S1 — Primers for ChIP-qPCR and RT-qPCR. (PDF) [file pgen.1002301.s017.pdf]

# Table S1 : Primer pairs for qPCR (ChIP and RT)

| **Primers for qpcr (ChIP)** | Primer name | | |
|---|---|---|---|
| ATGGATGGACAGTGTTCCTG | K20me3_F | | |
| CGTGTGCATGTGTGTACGTC | K20me3_R | | |
| AAAGCTGCCTGTAACACCTG | NegReg_F | | |
| TCCATCATGCTTTGGAGACC | NegReg_R | | |
| GATACATGCTCACGTCCTTGTC | cdx2_F | ChIP Controls | |
| TTCAACGTTTGTCCCCAGAC | cdx2_R | | |
| AGAGAGGGAGGAGGGGAAATG | gapdhF | | |
| AACAGGGAGGAGCAGAGAGCAC | gapdhR | | |
| GCTCCTGAAGATGTAAGCAATAAAG | IAP F | | |
| CTTCCTTGCGCCAGTCCCGAG | IAP R | | |
| GCACGTGTGCAGATTATGTTTAC | 4037_R_F | Full site right side | |
| CACTGTTTTGGTTTGTGGATG | 4037_R_R | | Empty site |
| CCAGTGCCCTAATTACCCAAC | J1_4037_F | | |
| TGCCCTAATTACCCAACCAC | 4037_L_F | Full site left side | |
| CCGGTTTGTTGTGTTCTTCC | 4037_L_R | | |
| CAGAGTGAGGGAAACAATGC | 4037_L_F2 | Left side - FAR | |
| GCCTGGACTATATTGCCACATC | 4037_L_R2 | | |
| TACTGCCCCATTTCTGCTTC | 4037_R_F2 | Right side - FAR | |
| TCATCCCTACCCCAGTTTTC | 4037_R_R2 | | |
| ATCTTCTGACGGCGAATGTG | 5338_R_F | Full site right side | |
| TCACTTGGAGTCAGGGACAAG | 5338_R_R | | |
| CAGAGCTAAGAAAAGGCTCCAG | J15338_R | | Empty site |
| CATTGTCCTTCTCCTGGTCAC | 5338_L_F | Full site left side | |
| CCGCAGAAGATTCTGGTTTG | 5338_L_R | | |
| TGGTGTTGAAGGAACAGTGC | 5338_L_F2 | Left side - FAR | |
| ATGGCAGTGACACCAAAGTG | 5338_L_R2 | | |
| TGCTGCATCCTCTGAAAGTC | 5338_R_F2 | Right side - FAR | |
| TGCCTATCCATGCACTTCTC | 5338_R_R2 | | |
| CGTGAACGCGTGTAAGAATC | 8818_R_F | Full site right side | |
| AAAGCCATATGAGGGCAGTG | 8818_R_R | | Empty site |
| CTGAGGGAAGATCTCCAGTTTC | J1_8818_F | | |
| TCAAACGTTCCCTGCCTAAC | 8818_L_F | Full site left side | |
| GGCTTCCCACAGTAGATGAAAC | 8818_L_R | | |
| TTTCTCAGGCCAACATTTCC | 8818_R_F2 | Left side - FAR | |
| AGGGTGAGACAGCTCTTTTCAG | 8818_R_R2 | | |
| CTGTGATTTCACCACTCAGGTC | 8818_L_F2 | Right side - FAR | |
| TCCAGAAAGGGAGAGATTCC | 8818_L_R2 | | |
| TTGCTCTTGCACTCTTGCTC | 8852_R_F | Full site right side | |
| GGGTGGGTTTATTCTTTGTCC | 8852_R_R | | Empty site |
| GGGACTCCTTTGACCATTTG | 8852_L_F | Full site left side | |
| AGGATGTTAGCGCCATCTTG | 8852_L_R | | |
| AGGAAAACCATGCCTCTGTG | 8852_L_F2 | Left side - FAR | |
| CTGCACATTATAAAGAGGGGAAG | 8852_L_R2 | | |
| TGTGTGTGAATGCCTCCTTC | 8852_R_F2 | Right side - FAR | |
| CATGTTGGAAAACCAGTAGGG | 8852_R_R3 | | |
| GGGCTAATTCCTTCTACCTTCC | 2754LF | Full site left side | |
| CTGGTCTGTTGTGTTCTTCCTG | 7305F | | |
| GCCTTTCATCTAAGTGCTGGTC | 2754RR | Full site right side | |
| CCAACACATTCTGAGTTGCAG | 2754RF | | |
| GGGCTAATTCCTTCTACCTTCC | J1-2754F | Empty site | |
| CTTCCACTGCAACTCAGAATG | J1-2754R | | |
| CAAAGGGTATGGGCTTTGC | 2754 LF-2 | Left side - FAR | |
| TCTCAACTCGTTCTCTCAGCAG | 2754 LR-2 | | |
| TCAACAGGTGTTTGCTAGGC | 2754 RF-2 | Right side - FAR | |
| TGAAAGCAGCTGTGAAATGG | 2754 RR-2 | | |
| AAGTAGAACATCGGTGCCAAC | IAPB3GAL_F | | |
| CACCCTTACTGTCCTCAAAGTG | IAPB3GAL_R | *b3galtl* analysis | |
| AGTTTGTCAGAGGGTCAGAAAAG | PromoB3bis_F | | |
| GCGGTTGTAGGTGGTCCTG | PromoB3bis_R | | |

| **Primers for qpcr (RT)** | Primer name |
|---|---|
| TGGGACTATGGACTCCGTTC | Tubb5-F |
| AAAGCCTTGCAGGCAATCA | Tubb5-R |
| GCTAGGTTTCTGCGGTCGCGTC | TBP F |
| CTGTACTGAGGCTGCTGCAGTTGCTAC | TBP R |
| TCACCAAGGGTGTGAGAAGTC | actin_F |
| TGGCCTTTTTAGGGACTCTG | actin_R |
| AAGCACGAGATTGCCCTCTA | b3galtl F |
| ACTCCACAGAGCGGTAGGAA | b3galtl R |
