# Supplementary material for: Retrotransposon-Induced Heterochromatin Spreading in the Mouse Revealed by Insertional Polymorphisms
Source: PLoS Genet. 2011 Sep 29;7(9):e1002301. doi: 10.1371/journal.pgen.1002301 (PMC3183085; doi:10.1371/journal.pgen.1002301)
Supplement: Text S1 — ETn/MusD elements are variably regulated by epigenetic mechanisms. References cited are [79], [80]. (PDF) [file pgen.1002301.s018.pdf]

# Text S1. ETn/MusD elements are variably regulated by epigenetic mechanisms.

Recombination between LTRs can create solo-LTRs which may be differently recognized by the genome compared to full-length copies. When observed separately, the H3K9me3 RPKM asymmetry between ETn/MusD full-length and solo-LTR copies is indeed different. While polymorphic solo-LTR sites maintain a distribution around 0 RPKM asymmetry, full-length polymorphic sites have a tendency towards more H3K9me3 when an ETn/MusD copy is present (Figure S6). Such skewing remains however less evident than that observed for IAP polymorphic copies (Figure 1C). MusDs have lower copy number than ETns and are also thought to be an older family [79]. Furthermore, MusD elements are likely less polymorphic than ETn since nearly all newly described mutational insertions of the ETn/MusD family are caused by ETns, rather than MusDs [37]. Hence, our set of polymorphic ETn/MusD copies is biased towards an overrepresentation of ETn copies. As we already established [37], at least the most active ETn subfamily, ETnII, is less marked with H3K9me3 than MusDs, and we confirm such tendency with 5 MusDs out of 6 inducing H3K9me3-heterochromatin while only 14 ETns out of 22 show skewing (Figure S6). The reason why ETns are able to induce H3K9me3-heterochromatin in some cases is unknown, but it does not seem to be related to sequence specificity. For polymorphic full-length ETn/MusD copies no influence of age, as described before [80], was observed but since the copies are polymorphic, a bias exists towards recent insertions. Furthermore, variation of DNA methylation on ETn copies is known [80] suggesting that the ETn/MusD family is a sporadic target of the epigenetic mechanisms of the host genome.
