# Supplementary material for: Retrotransposon-Induced Heterochromatin Spreading in the Mouse Revealed by Insertional Polymorphisms
Source: PLoS Genet. 2011 Sep 29;7(9):e1002301. doi: 10.1371/journal.pgen.1002301 (PMC3183085; doi:10.1371/journal.pgen.1002301)
Supplement: Text S2 — New putative IAP copies in ES cell lines. References cited are [81], [82]. (PDF) [file pgen.1002301.s019.pdf]

Text S2. New putative copies in ES cell lines.

Since we were able to correlate IAP polymorphic copies with H3K9me3 chromatin, we wondered if any region enriched in this repressive mark in only one of the cell lines studied could be the result of a new IAP insertion. We selected therefore all the H3K9me3 enriched regions present in J1 and absent in TT2 that could not be explained by any already described ERVs in the sequenced mouse genome or by our polymorphic ERV data set [30]. We found 201 regions only enriched in J1 compared to TT2. By comparing the reference B6 genome over the 201 regions with three 129 strains (129P2, 129S1/SvImJ, 129S5) sequenced in the Mouse Genomes Project (http://www.sanger.ac.uk/mousegenomes), we were able to attribute three new undescribed IAP copies to our regions, which were further confirmed through PCR and sequencing (data not shown).

Materials and Methods

For the 201 regions, IAP elements present in the three 129 strains (129P2, 129S1/SvImJ, 129S5) relative to the C57B6/J reference were identified using RetroSeq [unpublished], which looks for clusters of reads with one end mapping to the reference and the other mapping to a set of IAP probes obtained from Repbase [81]. RetroSeq requires that the anchoring read have a minimum MAQ mapping quality of 30 and at least 10 independent read pairs were required to support a call. Alignments to Repbase were performed with SSAHA2 [82] with a minimum of 90% identify and hit length of 36bp for a match.
